# Total Synthesis of (−)-Illisimonin A Enabled by Pattern Recognition and Olefin Transposition

**DOI:** 10.1021/jacs.5c05409

**Published:** 2025-05-13

**Authors:** Bo Xu, Ziyao Zhang, Mingji Dai

**Affiliations:** † Department of Chemistry, 1371 Emory University, Atlanta, Georgia 30322, United States; ‡ Department of Pharmacology and Chemical Biology, School of Medicine, Emory University, Atlanta, Georgia 30322, United States

## Abstract

We report an asymmetric total synthesis of (−)-illisimonin A, a sesquiterpene natural product with neurotrophic activity. Illisimonin A possesses a unique cage-like 5/5/5/5/5 pentacyclic scaffold containing a *trans*-pentalene and a norbornane, two highly strained and challenging structural motifs. It also contains seven contiguous fully substituted stereocenters, including three all-carbon quaternary centers, two of which are adjacent. Our synthesis exploits a pattern recognition strategy to identify a 5,6-fused bicyclic intermediate derived from (*S*)-carvone in two steps as the starting point and leverages five sequential olefin transposition reactions to decorate the bicyclic carbocycle. Other key steps include a tandem Mukaiyama hydration-translactonization to form the γ-butyrolactone and an intramolecular aldol cyclization to close the cage and finally deliver (−)-illisimonin A in 16 steps.

## Graphical abstract

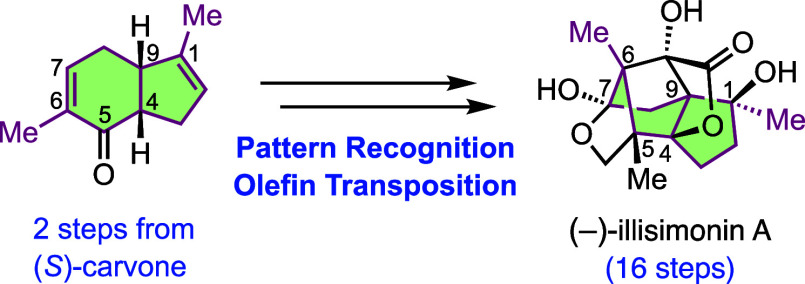

In 2017, Yu and co-workers isolated (−)-illisimonin A (**1**, Figure 1A) from the fruits of *Illicium simonsii*, an evergreen shrub often used in traditional Chinese medicine.^1^ The chemical structure of illisimonin A was identified to contain an unprecedented cage-like 5/5/5/5/5 pentacyclic scaffold. Within this highly congested ring system, there exists a strained *trans*-pentalene motif (highlighted in yellow), which is extremely rare in natural products and poses a significant challenge for chemical synthesis.^2^ In addition, there are seven contiguous fully substituted stereocenters on illisimonin A’s 15-carbon only skeleton. Three of these seven stereocenters are all-carbon quaternary centers (C5, C6, and C9), two of which are adjacent to each other. Biosynthetically, farnesyl diphosphate (**2**) was proposed as a key linear intermediate to construct the daunting architecture of illisimonin A via a series of enzymatic cyclizations and rearrangements (the cyclase phase) and oxidations (the oxidase phase) by going through the scaffolds of bisabolane (**3**), acorane (**4**), cedrane (**5**), and *allo*-cedrane (**6**).^1^ Recent computational investigations by McCulley and Tantillo revealed that the rearrangement of *allo*-cedrane to the illisimonane backbone (**7**) would require certain oxidation patterns (for example: X = OH) to lower the energy barrier of the last rearrangement (**6** to **7**).^3^ While the isolation yield is extremely low (∼0.000004%), Yu and co-workers managed to get 4 mg of illisimonin A from 96 kg of the *Illicium simonsii* fruits for structural characterization and preliminary biological evaluation. The latter revealed that illisimonin A has neuroprotective effects against oxygen-glucose deprivation-induced cell injury in SH-SY5Y cells (EC_50_ = ∼28 μM), warranting further investigation.^1^

**Figure 1:**
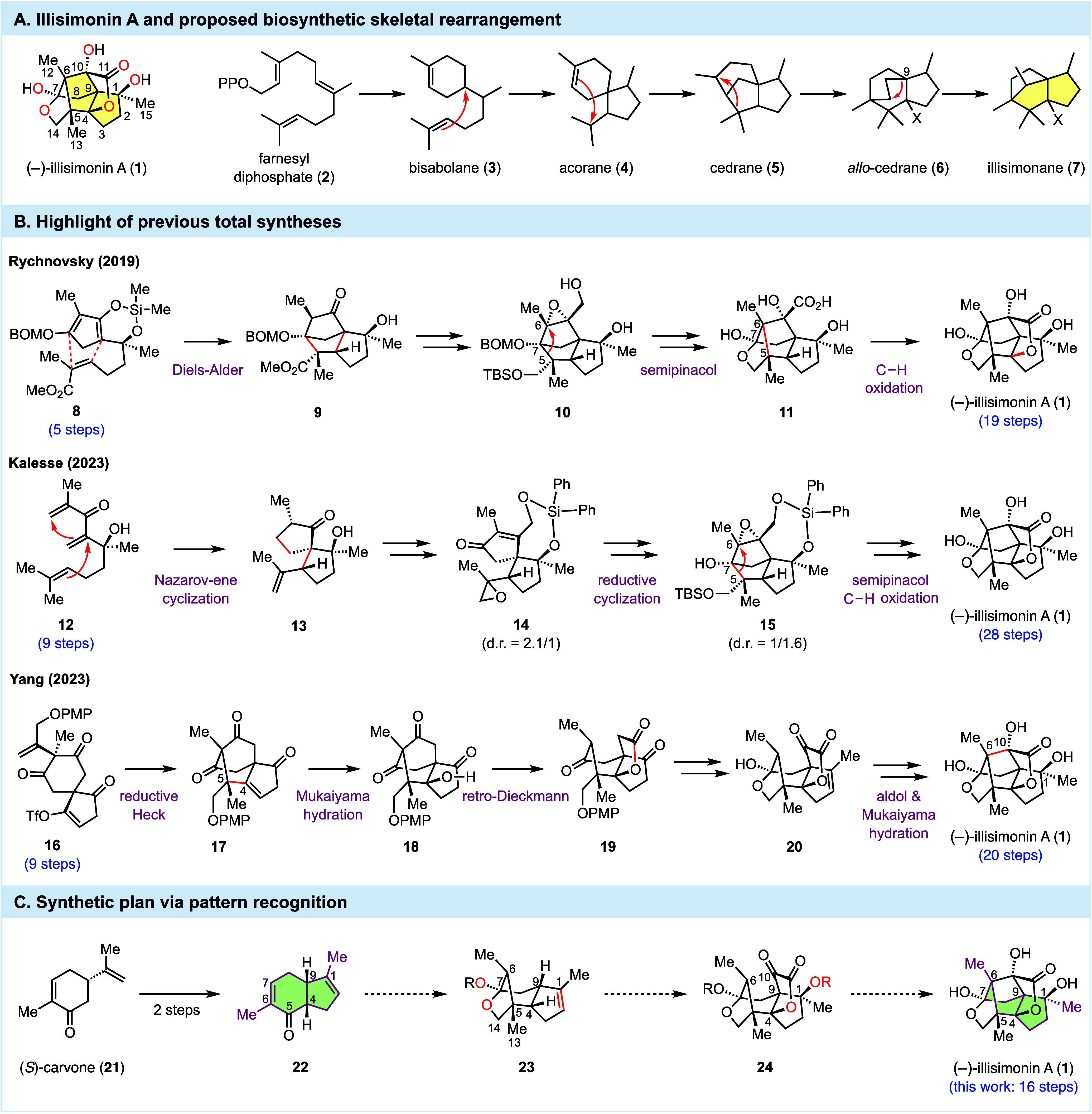
Structure, plausible biosynthesis, prior total syntheses, and our synthetic plan of (−)-illisimonin A.

The *Illicium* sesquiterpenes have garnered a significant amount of attention from the synthetic and medicinal communities.^4^ The unique bond linkage between C6 and C10 distinguishes illisimonin A from all the other *Illicium* sesquiterpenoid natural products including the famous anisatin,^5^ majucin,^6^ merrilactone,^7^ jiadifenolide,^8^ and 11-*O*-debenzoyltashironin.^9^ Similar to the aforementioned family members, illisimonin A has attracted chemists’ attention because of its complex and appealing topological architecture, therapeutic potential, and isolation burden (Figure 1B). In 2019, Rychnovsky and Burns reported the first total synthesis of illisimonin A.^10^ Their synthesis features a silyl ether-templated Diels–Alder reaction (**8** to **9**), a semipinacol rearrangement (**10** to **11**), and an iron-catalyzed C–H oxidation (**11** to **1**) to reach (±)-illisimonin A in 17 steps and (−)-illisimonin A in 19 steps. They also revised the absolute configuration of the natural product. In 2023, Kalesse and co-workers reported the second total synthesis of (−)-illisimonin A.^11^ Notable steps in their synthesis include a remarkable tandem Nazarov-ene cyclization to convert **12** to **13** and a reductive ketone-epoxide ring closure to build the C5–C7 linkage. Similar to the Rychnovsky synthesis, a semipinacol rearrangement and C–H oxidation were employed to form key ring systems and eventually deliver (−)-illisimonin A in 28 steps. In the same year, Yang and co-workers reported another total synthesis of (−)-illisimonin A.^12^ Their synthesis features an intramolecular Heck reaction to install the C5 all-carbon quaternary center (**16** to **17**). After Mukaiyama hydration, a retro-Dieckmann reaction was employed to convert **18** to **19**. Toward the end, they used an intramolecular aldol reaction to forge the C6–C10 linkage and another Mukaiyama hydration to install the C1 tertiary alcohol, completing their total synthesis in 20 steps. Additionally, Yang and co-workers proposed a different biosynthetic pathway from the cedrane skeleton to illisimonin A. Overall, these collective efforts highlight the ingenuity required to address the synthetic challenges posed by the complex architecture of illisimonin A and laid a strong foundation for continued innovation in chemistry.

Our long-term interest^13^ in natural products with neurotrophic activity^14^ led us to illisimonin A. Using a pattern recognition strategy (Figure 1C),^15^ we extracted the *cis*-hydrindane moiety of illisimonin A (**1**, highlighted in green) and mapped it onto 5,6-fused bicyclic compound **22**, an intermediate we prepared in two steps from (*S*)-carvone and used in our crinipellin total synthesis.^16^ We wondered about the possibility of using **22** as the starting point to synthesize (−)-illisimonin A. In our generic synthetic plan, we proposed to utilize the enone functionality in the six-membered ring to functionalize C5 by stereoselective formation of the all-carbon quaternary center and C7 by oxidizing it to the ketone oxidation level, which would then cyclize with the hydroxyl group at C14 to form acetal **23**. We next needed to properly functionalize the five-membered ring of **23** by selective oxidations at C1 and C4 and building an all-carbon quaternary center at C9. However, there is only an alkene functional group in the five-membered ring. How to orchestrate different alkene chemistry to precisely functionalize those positions would be critical to the success of our synthesis. If we could advance **23** to α-keto-γ-butyrolactone **24**, an intramolecular aldol reaction similar to Yang’s synthesis could be exploited to forge the C6–C10 linkage. However, we were not sure how the C1 tertiary alcohol would complicate the desired aldol cyclization because a retro-aldol reaction to break the C1–C9 bond or a translactonization could be potential competing pathways. Overall, the proposed approach would allow us to bypass the problems involved in directly forming the highly strained *trans*-pentalene motif and use **22**, a readily available intermediate prepared in 2 steps from (*S*)-carvone as the foundation of the entire synthesis. The obvious challenge lies in how to navigate the murky waters from **22** to (−)-illisimonin A. Herein, we report the details of our (−)-illisimonin A total synthesis from **22** in 14 steps (16 steps from (*S*)-carvone).

As shown in Scheme 1, following our previously established procedure,^16^ compound **22** was prepared from (*S*)-carvone (**21**) in two steps, namely, stereoselective α-allylation and ring closing metathesis followed by a one-pot epimerization. To oxidize C7, selective nucleophilic enone epoxidation with H_2_O_2_ and NaOH gave **25** in 77% yield as a single stereoisomer. To add the C14 carbon, a one-carbon Wittig homologation of the ketone was used to afford an enolether, which was especially unstable due to the existence of the epoxide and underwent hydrolysis and epoxide ring opening during aqueous quench and workup to provide aldehyde **26** in 91% yield. To install the C5 all-carbon quaternary center in a stereoselective manner, deconjugative α-methylation with MeI and *t*-BuOK was employed. The α-methylation occurred from the less hindered convex face. Meanwhile, *O*-methylation at the C7 secondary alcohol also took place to give a methyl ether. A subsequent aldehyde reduction in the same pot furnished **27** in 72% yield. We then envisioned a redox neutral and atom economic exomethylene isomerization to generate an enol ether, which could further react with the C14 primary alcohol to form an acetal at C7. This tandem process was achieved by treating **27** with the Crabtree catalyst (3 mol %) in refluxing THF.^17^ This highly efficient olefin transposition not only adjusted the oxidation level of C7 but also formed the first bridged ring system with the desired stereoconfiguration at C6. Compound **28** was produced in 65% yield on gram scale.

**Scheme 1:**
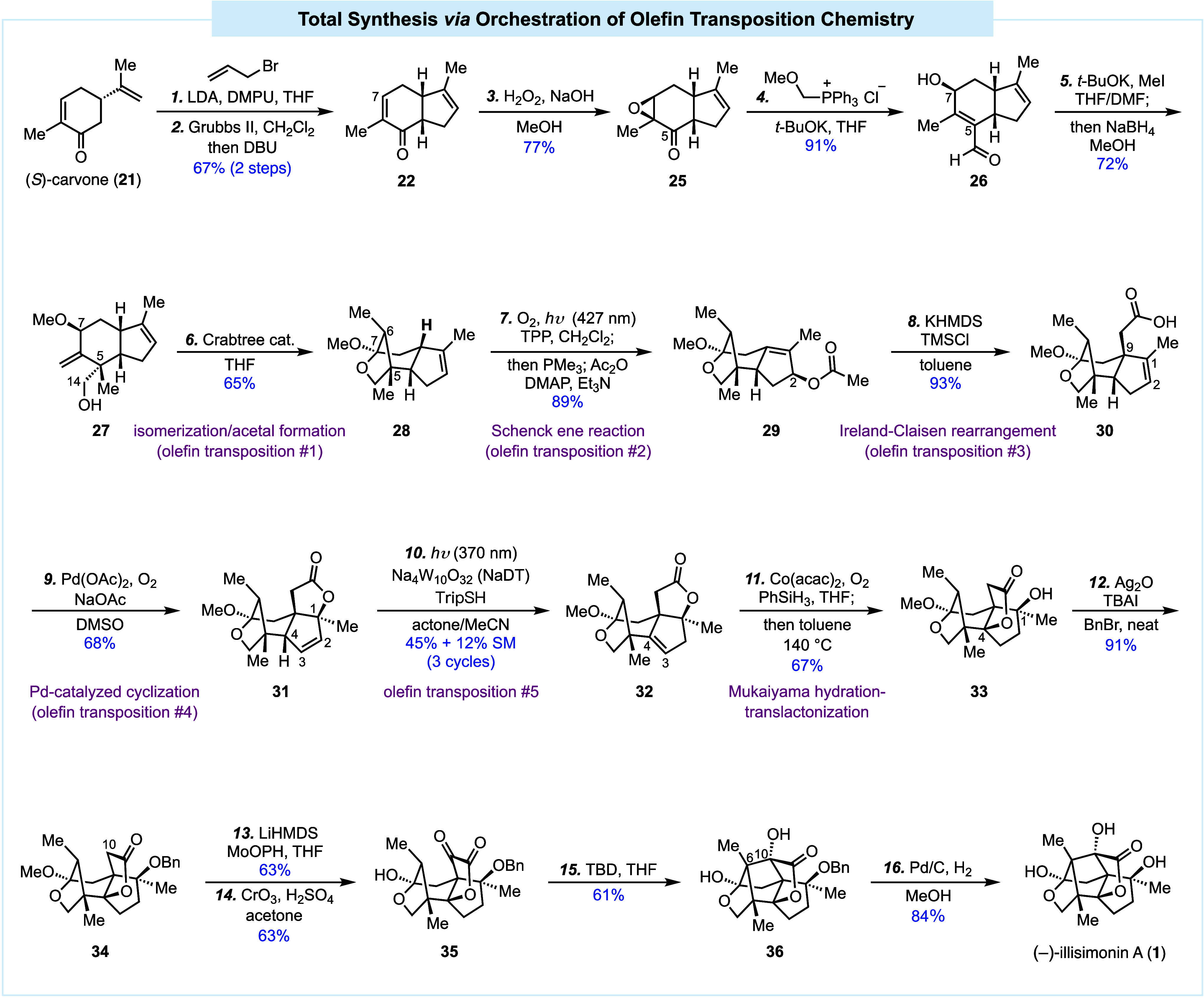
Total Synthesis of (−)-Illisimonin A

With the bridged acetal moiety constructed, we next focused on precise functionalization of the cyclopentene ring. While we were struggling with various unfruitful explorations, we noted that **28** was slowly oxidized to an allylic alcohol under air, presumably via a Schenck singlet oxygen ene reaction. We then decided to accelerate this process and irradiated (427 nm) **28** in the presence of oxygen and a catalytic amount of tetraphenylporphyrin (TPP). To our delight, after the newly formed peroxide was quenched with PMe_3_, followed by a one-pot acetate formation, allylic acetate **29** was obtained in 89% yield. This second olefin transposition is strategically important for our synthesis because it enabled a subsequent Ireland-Claisen rearrangement (**29** to **30**, the third olefin transposition) to install the C9 all-carbon quaternary center and the requisite carboxylic acid in 93% yield. The carboxylic acid was then used as a handle to introduce the C1 oxygen functionality via a palladium-catalyzed oxidative lactonization developed by Larock and co-workers.^18^ Lactone product **31** was produced in a 68% yield. This palladium-catalyzed cyclization further migrated the olefin (the fourth olefin transposition) from C1–C2 to C2–C3 as a consequence of a selective β-hydride elimination. This transposition renders the C4 position more reactive due to its allylic nature and opens an opportunity for direct allylic C–H hydroxylation reactions to install the missing tertiary alcohol. Unfortunately, we were unable to achieve such direct C–H hydroxylation, presumably due to the severe steric hindrance at this bis neopentyl position. We then envisioned a thermodynamically controlled positional olefin isomerization (the fifth olefin transposition) to convert the disubstituted C2–C3 olefin to the more stable trisubstituted C3–C4 olefin. This seemingly straightforward isomerization turned out to be very challenging, again due to steric hindrance. After unfruitful attempts with transition-metal-catalyzed double bond isomerizations, we took course to the recently developed photochemical positional alkene isomerization developed by Wendlandt and co-workers.^19^ While in the Wendlandt case a contrathermodynamic isomerization was achieved with the cooperative effect of decatungstate and cobaloxime catalysts, we believed that, if we could hijack the decatungstate-mediated hydrogen atom abstraction part of their catalytic process to generate an allylic radical, a thermodynamic isomerization from **31** to **32** might be achieved. To our delight, the desired product **32** was obtained with the Wendlandt conditions but at low conversion (∼20%). We further learned that the cobaloxime co-catalyst was not necessary in our case since we were trying to realize a thermodynamic isomerization. Further optimization led us to photochemical isomerization conditions similar to the one used by Xu and co-workers in their functional-group translocation chemistry.^20^ Eventually, with a combination of NaDT, TripSH, and light (370 nm) in a mixture of acetone/MeCN, the desired product **32** could be obtained in 45% yield after three cycles of isomerization, realizing the fifth olefin transposition in our synthesis.

With the olefin in the right position, compound **32** was subjected to a Co-catalyzed Mukaiyama hydration^21^ to install the desired hydroxyl group at C4. A subsequent one-pot thermal translactonization produced **33** in 67% yield. At this stage, the free tertiary alcohol of **33** was protected as a benzyl ether, because it did complicate the proposed aldol cyclization. Compound **34** was obtained in a 91% yield. The subsequent C10 oxidation was achieved with a combination of the MoOPH α-hydroxylation and Jones oxidation. The latter also hydrolyzed the acetal to the desired hemiacetal to give compound **35**. TBD-mediated aldol cyclization was next used to forge the C6–C10 linkage and convert **35** to **36** in a 61% yield. The final removal of the benzyl group with Pd/C and H_2_ advanced **36** to (−)-illisimonin A (**1**) and completed our 16-step total synthesis.

In summary, using pattern recognition analysis, we traced (−)-illisimonin A back to bicyclic intermediate **22**, which can be synthesized in two steps from (*S*)-carvone. We utilized the enone functionality of **22** to install the C5 all-carbon quaternary center and adjust the oxidation state at C7 via a sequence of nucleophilic epoxidation, tandem Wittig homologation and hydrolysis, and deconjugative alkylation. Five olefin transposition reactions including a tandem exomethylene-enol ether isomerization and acetal formation, a Schenck singlet oxygen ene reaction, an Ireland-Claisen rearrangement, a Pd-catalyzed oxidative cyclization, and a decatungstate-catalyzed photochemical positional alkene isomerization were then orchestrated to precisely functionalize the fused bicyclic carbocyle. Toward the end, an intramolecular aldol reaction was used to close the cage-like ring system. Overall, with these enabling synthetic transformations, we assembled the entire chemical architecture of (−)-illisimonin A in 16 steps from (*S*)-carvone.

## Supplementary Material



## Notes

Experimental procedures and NMR spectra for all new compounds (PDF)

## Abbreviations

DMPU: *N*,*N*′-dimethylpropyleneurea
THF: tetrahydrofuran
KHMDS: potassium bis­(trimethylsilyl)­amide
LDA: lithium diisopropylamide
TPP: tetraphenylporphyrin
DMAP: 4-dimethylaminopyridine
TBAI: tetra-*n*-butylammonium iodide
DBU: 8-diazabicyclo­[5.4.0]­undec-7-ene
DMSO: dimethyl sulfoxide
TBD: 1,5,7-triazabicyclo­[4.4.0]­dec-5-ene
DMF: dimethylformamide
TripSH: triisopropylsilanethiol
MoOPH: oxodiperoxymolybdenum­(pyridine)-(hexamethyl phosphoric triamide)
TMSCl: trimethylsilyl chloride
