# Supplementary material for: Total Synthesis of (−)-Illisimonin A Enabled by Pattern Recognition and Olefin Transposition
Source: J Am Chem Soc. 2025 May 13;147(21):17592–7. doi: 10.1021/jacs.5c05409 (PMC12123608; doi:10.1021/jacs.5c05409)
Supplement: Supplementary file 1 [file ja5c05409_si_001.pdf]

**Supporting Information**

# Total Synthesis of (−)-Illisimonin A Enabled by Pattern Recognition and Olefin Transposition

Bo Xu,[1] Ziyao Zhang,[1] Mingji Dai*,[1,2]

[1]Department of Chemistry, Emory University, Atlanta, Georgia 30322, United States;

[2]Department of Pharmacology and Chemical Biology, School of Medicine, Emory University, Atlanta, Georgia 30322, United States.

## Table of Content

*A. General Methods.* ........................................................................................................ 3

*B. Experimental Procedures for the Total Synthesis of Illisimonin A* ........................................ 4

*C. NMR Comparison Tables* ................................................................................................ 15

*D. $^{1}H$ and $^{13}C$ NMR spectra* ............................................................................................. 19

**A. General Methods.** All commercially available compounds were purchased from Sigma-Aldrich, Alfa-Aesar, Oakwood chemicals and Ambeed unless otherwise noted. Materials obtained from commercial suppliers were used without further purification. NMR spectra were recorded on Bruker spectrometers ($^{1}H$ at 400 MHz, 600 MHz, 800 MHz and $^{13}C$ at 100 MHz, 150 MHz, 200 MHz). Chemical shifts (δ) were given in ppm with reference to solvent signals [$^{1}H$ NMR: $CDCl_3$ (7.26); $^{13}C$ NMR: $CDCl_3$ (77.16); $^{1}H$ NMR: Methanol-$d_4$ (3.31); $^{13}C$ NMR: Methanol-$d_4$ (49.00).]. $^{1}H$ NMR data are reported as follows: chemical shift (δ ppm), multiplicity (s = singlet, d = doublet, t = triplet, q = quartet, m = multiplet, br = broad, app = apparent), coupling constant (Hz), and integration. IR spectra were collected on a Nicolet iS10 FT-IR spectrometer. Mass spectra were taken on a Thermo Finnigan LTQ-FTMS spectrometer with APCI, ESI. Optical rotation were determined by Autopol IV(Rudolph Research Analytical). Column chromatography was performed on silica gel. All reactions sensitive to air or moisture were conducted under argon atmosphere in dry solvents under anhydrous conditions, unless otherwise noted. Dry THF and DCM ($CH_2Cl_2$) were processed via PureProcessTechnology GS-SPS-5-CM system. Dry $Et_2O$ and toluene were purchased from Sigma-Aldrich. All other solvents and reagents were used as obtained from commercial sources without further purification. Room temperature (r.t.) is around 23 °C.

Abbreviation table

| Abbreviation | Full name |
|---|---|
| THF | Tetrahydrofuran |
| DMF | *N*,*N*-Dimethylformamide |
| TPP | Tetraphenylporphyrin |
| DMAP | 4-Dimethylaminopyridine |
| KHMDS | Potassium hexamethyldisilazide |
| TripSH | Triisopropylsilanethiol |
| TBAI | Tetra-*n*-butylammonium iodide |
| LiHMDS | Lithium hexamethyldisilazide |
| MoOPh | Oxodiperoxymolybdenum(pyridine)-(hexamethylphosphoric triamide) |
| TBD | Triazabicyclodecene |

## B. Experimental Procedures for the Total Synthesis of Illisimonin A

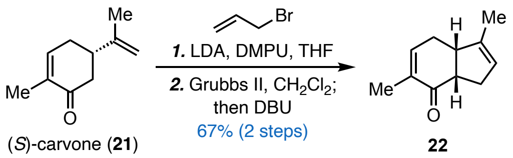


For the synthesis of known compound **22**, see: Xu, B.; Zhang, Z.; Tantillo, D. J.; Dai, M. Concise Total Syntheses of (−)-Crinipellins A and B Enabled by a Controlled Cargill Rearrangement. *J. Am. Chem. Soc.* **2024**, *146*, 21250-21256.

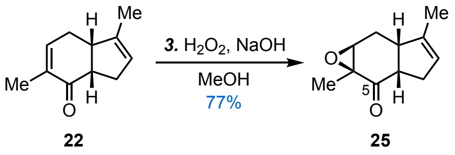


Enone **22** (9.0 g, 55.48 mmol, 1.0 equiv) was dissolved in 300 mL of methanol in a 500 mL round-bottom flask. The mixture was cooled to – 20 °C before aqueous NaOH solution (4.0 M, 4.2 mL, 16.8 mmol, 0.3 equiv) was added, followed by the dropwise addition of a 30% $H_2O_2$ solution (6.8 mL, 66.57 mmol, 1.2 equiv). The reaction mixture was stirred overnight at this temperature. After completion, the reaction mixture was quenched with saturated ammonium chloride and extracted with hexanes and the combined organic layers were washed with aq. sat. $Na_2SO_3$ and brine, dried over $Na_2SO_4$, and concentrated under reduced pressure. The crude product was purified by flash chromatography (eluent: hexanes to hexanes/EtOAc = 24:1) to afford epoxide **25** (7.66 g, 43.0 mmol, 77%) as a pale yellow oil.

**$[\alpha]_D^{20}$** = –31.7 ($c$ = 0.3, $CHCl_3$).

**IR (neat):** 2966, 2929, 2861, 2361, 1702, 1438, 1378, 1348, 1275, 1259, 1092, 1050 $cm^{-1}$.

**$^1$H NMR (600 MHz, $CDCl_3$):** δ 5.23 (dq, $J$ = 2.9, 1.5 Hz, 1H), 3.39 (d, $J$ = 4.1 Hz, 1H), 3.21 – 3.12 (m, 2H), 2.74 – 2.62 (m, 1H), 2.55 – 2.44 (m, 2H), 1.65 (dq, $J$ = 2.3, 1.1 Hz, 3H), 1.53 – 1.47 (m, 1H), 1.47 (s, 3H).

**$^{13}$C NMR (151 MHz, $CDCl_3$):** δ 211.0, 141.2, 123.2, 62.3, 60.8, 47.6, 44.7, 33.2, 27.5, 15.2, 14.9.

**HRMS *m/z* (APCI):** calc. for $C_{11}H_{15}O_2^+$ $[M+H]^+$: 179.1067, found: 179.1066.

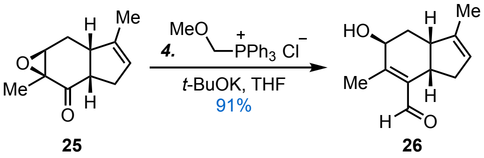


$MeOCH_2PPh_3$ chloride (60.6 g, 176.85 mmol, 2.0 equiv) was added to a 1 L round bottom flask. The flask was evacuated and backfilled with argon three times. Anhydrous THF (450 mL) was added. The solution

was cooled to 0 °C before *t*-BuOK (19.8 g, 176.85 mmol, 2.0 equiv) in 176 mL THF was added dropwise. The solution was stirred for 30 min at 0 °C then a solution of epoxide **25** (15.76 g, 88.43 mmol, 1.0 equiv) in THF 50 mL was added dropwise to the reaction mixture. The mixture was allowed to warm to room temperature, then heated to 60 °C for 3 h and then cooled to 0 °C before it was quenched with a solution of sat. aq. $NH_4Cl$ and extracted with $Et_2O$. The combined organic layers were washed with brine, dried over $Na_2SO_4$, and concentrated under reduced pressure. The crude product was subjected to flash chromatography (hexanes:EtOAc = 3:1 to 1:2) to yield enal **26** (15.47 g, 80.46 mmol, 91%) as a yellow oil.

**$[\alpha]_D^{20}$** = 82.9 ($c$ = 0.8, $CHCl_3$).

**IR (neat):** 3470, 2931, 2855, 2360, 1667, 1445, 1377, 1352, 1275, 1261, 1149, 1141, 1047, 1021 $cm^{-1}$.

**$^1$H NMR (600 MHz, $CDCl_3$):** δ 10.17 (s, 1H), 5.39 – 5.24 (m, 1H), 4.19 (dd, $J$ = 9.3, 4.7 Hz, 1H), 3.15 – 3.02 (m, 1H), 2.83 (ddt, $J$ = 16.1, 9.1, 2.2 Hz, 1H), 2.59 – 2.49 (m, 1H), 2.27 (t, $J$ = 1.3 Hz, 3H), 2.07 (dt, $J$ = 12.7, 4.7 Hz, 1H), 1.94 – 1.78 (m, 2H), 1.785 (dtd, $J$ = 2.5, 1.7, 0.8 Hz, 3H), 1.49 (ddd, $J$ = 12.7, 10.8, 9.2 Hz, 1H).

**$^{13}$C NMR (151 MHz, $CDCl_3$):** δ 192.6, 155.8, 143.0, 136.8, 124.0, 71.5, 44.4, 38.0, 36.8, 33.3, 15.0, 13.6.

**HRMS *m*/*z* (APCI):** calc. for $C_{12}H_{17}O_2^+$ $[M+H]^+$: 193.1223, found: 193.1223.

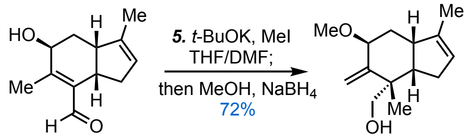


*t*-BuOK (7.56 g, 62.42 mmol, 3.0 equiv) was placed in a 250 mL round-bottom flask under an argon atmosphere, followed by the addition of 80 mL anhydrous THF. The reaction mixture was cooled to −78 °C before a solution of enal **26** (4.0 g, 20.81 mmol, 1.0 equiv) in 80 mL DMF was added dropwise. After stirring for 1 hour, MeI (5.2 mL, 83.22 mmol, 4.0 equiv) was added dropwise while ensuring efficient stirring. The reaction mixture was allowed to warm to room temperature and stirred for overnight. The mixture was then cooled to 0 °C before 80 mL methanol was added to quench the reaction. $NaBH_4$ (1.67 g, 44.11 mmol, 2.0 equiv) was then added in portions, and the mixture was stirred for 1 hour before being quenched with water. The aqueous layer was extracted with $Et_2O$, and the combined organic layers were washed with brine, dried over $Na_2SO_4$, and concentrated under reduced pressure. The crude product was subjected to flash chromatography (hexanes:EtOAc = 20:1 to 5:1) to yield alcohol **27** (3.34 g, 15.02 mmol, 72%) as a yellow oil.

**$[\alpha]_D^{20}$** = 10.3 ($c$ = 0.4, $CHCl_3$).

**IR (neat):** 3426, 2960, 2929, 2873, 1633, 1447, 1377, 1275, 1262, 1193, 1087, 1062, 1030 $cm^{-1}$.

**$^{1}$H NMR (600 MHz, CDCl$_3$):** δ 5.25 (dt, $J$ = 3.1, 1.6 Hz, 1H), 5.08 (p, $J$ = 0.6 Hz, 1H), 5.00 (d, $J$ = 1.3 Hz, 1H), 3.76 – 3.65 (m, 2H), 3.56 (d, $J$ = 10.7 Hz, 1H), 3.25 (s, 3H), 2.69 – 2.62 (m, 1H), 2.18 (dt, $J$ = 10.2, 7.2 Hz, 1H), 2.16 – 2.08 (m, 1H), 2.09 – 1.95 (m, 2H), 1.72 (ddt, $J$ = 2.7, 1.7, 0.8 Hz, 3H), 1.37 (ddd, $J$ = 14.0, 10.4, 3.6 Hz, 1H), 1.30 (s, 3H).

**$^{13}$C NMR (151 MHz, CDCl$_3$):** δ 149.0, 146.1, 122.9, 113.0, 82.9, 69.7, 55.9, 49.4, 41.9, 41.4, 33.5, 32.1, 25.2, 15.4.

**HRMS *m/z* (APCI):** calc. for $C_{14}H_{23}O_2^+$ [M+H]$^+$: 223.1693, found: 223.1693.

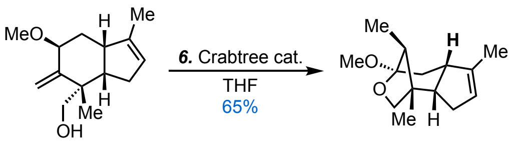


**27** **28**

Crabtree's catalyst (363 mg, 0.451 mmol, 0.03 equiv) was dissolved in 20 mL THF. The solution was stirred under $H_2$ atmosphere for 1 hour. The reaction mixture was degassed using three freeze-pump-thaw cycles. A solution of the alcohol **27** (3.34 g, 15.02 mmol, 1.0 equiv) in 100 mL of THF was then added to the prepared catalyst solution. A condenser was then attached to the flask, and the combined mixture was further degassed with an additional freeze-pump-thaw cycle. The reaction mixture was heated to reflux and stirred overnight. After completion, the mixture was concentrated under vacuum. The crude product was directly subjected to flash chromatography (eluent: hexanes to 3% EtOAc in hexanes) to yield ketal **28** (2.16 g, 9.72 mmol, 65%) as a colorless oil.

**$[\alpha]_D^{20}$** = –68.5 ($c$ = 0.1, CHCl$_3$).

**IR (neat):** 3041, 2960, 2930, 2864, 2362, 1460, 1442, 1307, 1275, 1235, 1211, 1195, 1181, 1166, 1143, 1125, 1091, 1081, 1068, 1046 cm$^{-1}$.

**$^{1}$H NMR (600 MHz, CDCl$_3$):** δ 5.36 – 5.24 (m, 1H), 3.64 (d, $J$ = 7.8 Hz, 1H), 3.47 (ddd, $J$ = 7.8, 1.6, 0.5 Hz, 1H), 3.38 (s, 3H), 2.91 (tdd, $J$ = 9.4, 3.1, 1.5 Hz, 1H), 2.50 (d, $J$ = 13.5 Hz, 1H), 2.48 – 2.38 (m, 1H), 2.29 (tt, $J$ = 8.3, 1.4 Hz, 1H), 2.12 (ddq, $J$ = 16.7, 3.2, 1.6 Hz, 1H), 1.74 (m, 3H), 1.71 (ddd, $J$ = 13.5, 9.3, 0.8 Hz, 1H), 1.64 (q, $J$ = 7.1 Hz, 1H), 0.95 (d, $J$ = 7.1 Hz, 3H), 0.88 (s, 3H).

**$^{13}$C NMR (150 MHz, CDCl$_3$):** δ 142.7, 122.6, 108.3, 70.9, 50.7, 49.5, 48.7, 47.1, 44.5, 34.2, 33.5, 17.7, 15.4, 8.7.

**HRMS *m/z* (APCI):** calc. for $C_{14}H_{23}O_2^+$ [M+H]$^+$: 223.1693, found: 223.1691.

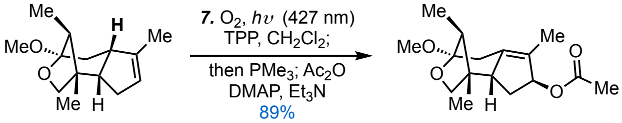


**28** **29**

Ketal **28** (3.81 g, 17.14 mmol, 1.0 equiv) and TPP (105 mg, 0.17 mmol, 0.01 equiv) were added to a 250 mL round-bottom flask. The flask was evacuated and backfilled with oxygen three times. Anhydrous $CH_2Cl_2$ (170 mL) was added, and the solution was cooled to −78 °C. The reaction mixture was irradiated with a 427 nm lamp until full conversion of ketal was detected by TLC. The flask was then evacuated and backfilled with argon three times. To the reaction mixture, $PMe_3$ (20.6 mL, 20.6 mmol, 1.2 equiv) in 1.0 M THF was added. The flask was then placed in an ice bath. After stirring for 30 minutes, DMAP (209 mg, 1.71 mmol, 0.1 equiv) and $Et_3N$ (7.2 mL, 51.41 mmol, 3.0 equiv) were added, followed by the dropwise addition of $Ac_2O$ (3.24 mL, 34.27 mmol, 2.0 equiv). The reaction mixture was stirred at 0 °C for 1 hour before it was quenched with saturated aqueous $NaHCO_3$. The organic phase was extracted with $Et_2O$. The combined organic layers were washed with brine, dried over $Na_2SO_4$, and concentrated under reduced pressure. The crude product was purified by flash chromatography (eluent: hexanes to 18% EtOAc in hexanes) to afford ester **29** (4.27 g, 15.23 mmol, 89%) as a white solid.

**$[\alpha]_D^{20}$** = –51.0 ($c$ = 1.0, $CHCl_3$).

**IR (neat):** 2968, 2916, 2873, 2837, 2362, 1728, 1482, 1448, 1371, 1348, 1316, 1279, 1235, 1205, 1191, 1168, 1139, 1119, 1088, 1080, 1031 $cm^{-1}$.

**$^1$H NMR (600 MHz, $CDCl_3$):** δ 5.52 (d, $J$ = 7.0 Hz, 1H), 3.50 (d, $J$ = 8.2 Hz, 1H), 3.44 (d, $J$ = 8.3 Hz, 1H), 3.3 (s, 3H), 2.99 (d, $J$ = 14.4 Hz, 1H), 2.81 (d, $J$ = 7.7 Hz, 1H), 2.12 (d, $J$ = 14.9 Hz, 1H), 2.03 (d, $J$ = 0.9 Hz, 3H), 1.85 (dq, $J$ = 9.6, 7.1 Hz, 2H), 1.81 – 1.73 (dt, $J$ = 14.7, 7.3 Hz, 1H), 1.68 (t, $J$ = 2.2 Hz, 3H), 0.9 (d, $J$ = 7.0 Hz, 3H), 0.89 (s, 3H).

**$^{13}$C NMR (151 MHz, $CDCl_3$):** δ 171.5, 141.7, 132.0, 109.0, 83.1, 71.1, 55.3, 51.1, 50.5, 46.1, 35.0, 32.5, 21.4, 16.8, 11.9, 8.9.

**HRMS *m/z* (APCI):** calc. for $C_{16}H_{25}O_4^+$ $[M+H]^+$: 281.1747, found: 281.1747.

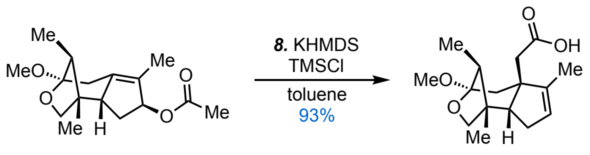


29 30

To a solution of ester **29** (714 mg, 2.547 mmol, 1.0 equiv) in 25 mL toluene, KHMDS (1.0 M in THF, 5.1 mL, 5.093 mmol, 2.0 equiv) was added slowly at −78 °C. After stirring at this temperature for 1 h, TMSCl (807 μL, 6.368 mmol, 2.5 equiv) was added. This mixture was allowed to warm to room temperature and stirred for another 1 h before heated to 80 °C and stirred at the same temperature overnight. After cooling down to room temperature, the reaction was quenched with 0.5 N aqueous HCl. The resulting mixture was extracted with EtOAc, and the combined organic layers were washed with brine, dried over $Na_2SO_4$ and concentrated under reduced pressure. The crude product was purified by flash chromatography (eluent:

hexanes to 15%, then 30% EtOAc in hexanes, then 100% EtOAc) to afford the carboxylic acid **30** (664 mg, 2.368 mmol, 93%) as a white-yellow solid.

**[*α*]$_D$$^{20}$** = –20.1 (*c* = 0.2, CHCl$_3$).

**IR (neat):** 3041, 2969, 2934, 2877, 1727, 1703, 1484, 1450, 1386, 1344, 1334, 1276, 1260, 1207, 1180, 1148, 1109, 1078, 1048 cm$^{-1}$.

**$^1$H NMR (600 MHz, CDCl$_3$):** δ 5.37 (dt, *J* = 3.3, 1.7 Hz, 1H), 3.59 (d, *J* = 7.9 Hz, 1H), 3.49 (dd, *J* = 7.9, 1.5 Hz, 1H), 3.38 (s, 3H), 2.58 (d, *J* = 13.5 Hz, 1H), 2.48 (ddt, *J* = 17.0, 7.4, 2.4 Hz, 1H), 2.45 – 2.34 (m, 2H), 2.21 (d, *J* = 14.4 Hz, 1H), 2.11 – 1.99 (m, 1H), 1.81 (q, *J* = 7.1 Hz, 1H), 1.77 (d, *J* = 13.5 Hz, 1H), 1.72 (dt, *J* = 3.0, 1.6 Hz, 3H), 0.95 (d, *J* = 7.1 Hz, 3H), 0.90 (s, 3H).

**$^{13}$C NMR (151 MHz, CDCl$_3$):** δ 177.6, 145.3, 123.9, 108.2, 71.3, 52.7, 51.2, 50.8, 48.4, 47.2, 44.3, 38.0, 32.4, 17.7, 13.8, 8.6.

**HRMS *m*/*z* (APCI):** calc. for $C_{16}H_{25}O_4^+$ [M+H]$^+$: 281.1747, found: 281.1746.

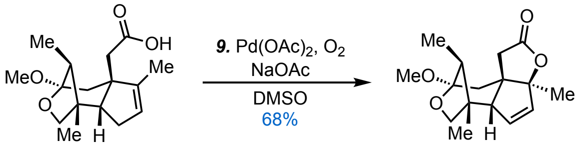


30 31

A flame dried 100 mL round-bottom flask was charged with acid **30** (660 mg, 2.354 mmol, 1.0 equiv), NaOAc (965 mg, 11.77 mmol, ) and Pd(OAc)$_2$ (264 mg, 1.177 mmol, 0.5 equiv) then DMSO (25 mL) was added. The flask was evacuated and backfilled with oxygen three times. This solution was stirred for 24 h at 90 °C. The mixture was then cooled to 0 °C and quenched with sat. aq. NH$_4$Cl, and extracted with Et$_2$O. The combined organic layers were washed with brine, dried over Na$_2$SO$_4$, and concentrated under reduced pressure. The crude product was subjected to flash chromatography (eluent: hexanes to 10%, then 20% EtOAc in hexanes) to yield lactone **31** (451 mg, 1.601 mmol, 68%) as a white solid.

**[*α*]$_D$$^{20}$**= 179.4 (*c* = 0.5, CHCl$_3$).

**IR(neat):** 3056, 2974, 2932, 2879, 2836, 2358, 2334, 1760, 1491, 1460, 1412, 1382, 1353, 1341, 1263, 1211, 1162,1183, 1134, 1110, 1098, 1975, 1047 cm$^{-1}$.

**$^1$H NMR (600 MHz, CDCl$_3$):** δ 6.01 (dd, *J* = 6.0, 1.7 Hz, 1H), 5.94 – 5.81 (m, 1H), 3.49 – 3.45 (m, 1H), 3.39 (d, *J* = 8.5 Hz, 1H), 3.38 (s, 3H), 2.94 (d, *J* = 14.3 Hz, 1H), 2.80 – 2.75 (m, 1H), 2.73 (dt, *J* = 3.0, 1.5 Hz, 1H), 2.62 (d, *J* = 18.1 Hz, 1H), 1.77 (q, *J* = 7.0 Hz, 1H), 1.61 (s, 3H), 1.56 (d, *J* = 14.3 Hz, 1H), 0.99 (d, *J* = 7.0 Hz, 3H), 0.97 (s, 3H).

**$^{13}$C NMR (151 MHz, CDCl$_3$):** δ 175.1, 137.6, 132.5, 107.7, 100.5, 70.0, 66.4, 51.6, 50.9, 49.1, 48.1, 46.5, 41.0, 22.6, 17.6, 8.4.

**HRMS *m*/*z* (APCI):** calc. for $C_{16}H_{23}O_4^+$ [M+H]$^+$: 279.1591, found: 279.1592.

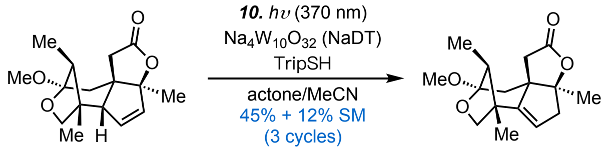


**31** **32**

Lactone **31** (200 mg, 0.717 mmol, 1.0 equiv), NaDT (35 mg, 0.0155 mmol, 0.02 equiv.), and TripSH (90 μL, 0.3583 mmol, 0.5 equiv) was added to a 25 mL flask, which was evacuated and backfilled with argon. MeCN/acetone (3 mL/3 mL) was then added as the solvent. The reaction mixture was degassed using four freeze-pump-thaw cycles to ensure the removal of oxygen. The reaction flask was placed under 370 nm LED Kessil PR160L lamps with a cooling fan, and the mixture was stirred at 1500 rpm. After stirring for 20 hours, the reaction mixture was filtered through a silica gel pad and washed with EtOAc. The combined filtrate was concentrated under reduced pressure. The crude product was purified by flash chromatography using a gradient eluent system (0% to 10% to 18% to 20% EtOAc in hexanes) to afford product **32** (46 mg, 0.323 mmol, 23%) as a white solid and recovered SM (101 mg, 0.362 mmol, 51%). The recovered starting material (101 mg) was subjected to the same procedure, yielding a new isomerized product (28 mg, 0.100 mmol, 28%) along with additional recovered SM (50 mg, 0.179 mmol, 50%). The recovered starting material (50 mg) was processed again following the same steps, yielding more of the desired product (15 mg, 0.054 mmol, 30%) and recovered SM (23 mg, 0.082 mmol, 46%). After three cycles, 89 mg of **32** (0.319 mmol, 45%) was obtained and 23 mg of starting material **31** (0.082 mmol, 12%) was recovered.

**$[\alpha]_D^{20}$** = 124.6 ($c$ = 0.1, $CHCl_3$).

**IR (neat):** 2959, 2925, 2889, 2854, 1765, 1463, 1411, 1386, 1356, 1342, 1263, 1211, 1164, 1182, 1134, 1112, 1099, 1075, 1054, 1045 $cm^{-1}$.

**$^1$H NMR (600 MHz, $CDCl_3$):** δ 5.52 (dd, $J$ = 3.1, 1.9 Hz, 1H), 3.81 (d, $J$ = 6.7 Hz, 1H), 3.41 (s, 3H), 3.34 (d, $J$ = 6.7 Hz, 1H), 2.84 (dd, $J$ = 17.5, 1.9 Hz, 1H), 2.79 (d, $J$ = 17.7 Hz, 1H), 2.65 (d, $J$ = 17.7 Hz, 1H), 2.56 (dd, $J$ = 17.4, 3.1 Hz, 1H), 2.48 (d, $J$ = 13.5 Hz, 1H), 1.94 (q, $J$ = 6.9 Hz, 1H), 1.85 (d, $J$ = 13.4 Hz, 1H), 1.46 (s, 3H), 1.17 (s, 3H), 0.95 (d, $J$ = 6.9 Hz, 3H).

**$^{13}$C NMR (151 MHz, $CDCl_3$):** δ 175.6, 156.2, 119.8, 108.9, 95.9, 78.2, 53.2, 50.8, 48.8, 47.0, 45.3, 43.6, 42.7, 22.9, 16.4, 8.0.

**HRMS *m/z* (APCI):** calc. for $C_{16}H_{23}O_4^+$ $[M+H]^+$: 279.1591, found: 279.1592.

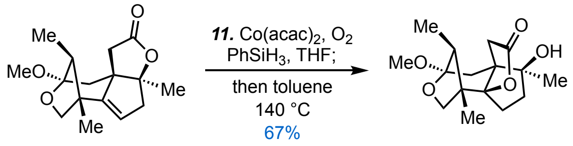


**32** **33**

A flask containing a mixture of lactone **32** (70 mg, 0.251 mmol, 1.0 equiv) and Co(acac)$_2$ (19 mg, 0.0754 mmol, 0.3 equiv) was evacuated and backfilled with oxygen for 3 times. THF (5 mL) was then used to dissolve this mixture. To this solution was added PhSiH$_3$ (270 uL, 1.506 mmol, 6.0 equiv). The reaction was stirred at 20 °C under $O_2$ atmosphere for overnight before it was concentrated. The resulting residue was dissolved in xylene and concentrated to remove low-boiling components. Toluene (5 mL) and NaHCO$_3$ (42 mg, 0.5 mmol, 2.0 equiv) were then added. The mixture was degassed and sealed in a microwave reaction vial and heated at 140 °C for overnight. The mixture was then cooled to room temperature and filtered through a silica gel pad. The silica gel pad was washed with EtOAc. The combined solution was concentrated under reduced pressure to afford the crude product, which was subjected to flash chromatography (eluent: 10% to 30% to 50% EtOAc in hexanes) to yield alcohol **33** (50 mg, 0.168 mmol, 67%) as a white solid.

**$[\alpha]_D^{20}$** = –25.1 ($c$ = 0.1, CHCl$_3$).

**IR (neat):** 2453, 2927, 2855, 1460, 1419, 1387, 1347, 1275, 1260, 1220, 1201, 1179, 1155, 1136, 1116, 1093, 1065 cm$^{-1}$.

**$^1$H NMR (600 MHz, CDCl$_3$):** δ 3.70 (d, $J$ = 9.7 Hz, 1H), 3.65 (d, $J$ = 9.7 Hz, 1H), 3.38 (s, 3H), 3.24 (d, $J$ = 18.6 Hz, 1H), 2.31 (d, $J$ = 18.6 Hz, 1H), 2.20 – 2.12 (m, 2H), 2.06 (ddd, $J$ = 14.3, 7.4, 4.5 Hz, 1H), 1.95 (d, $J$ = 14.4 Hz, 1H), 1.91 – 1.84 (m, 2H), 1.73 (ddd, $J$ = 13.2, 10.1, 7.4 Hz, 1H), 1.34 (s, 3H), 1.09 (s, 3H), 0.95 (d, $J$ = 6.9 Hz, 3H) .

**$^{13}$C NMR (151 MHz, CDCl$_3$):** δ 176.0, 107.6, 101.2, 81.9, 70.8, 52.3, 50.8, 50.7, 43.1, 42.3, 41.8, 38.6, 32.9, 23.5, 15.1, 8.5.

**HRMS *m/z* (APCI):** calc. for $C_{16}H_{25}O_5^+$ [M+H]$^+$: 297.1697, found: 297.1699.

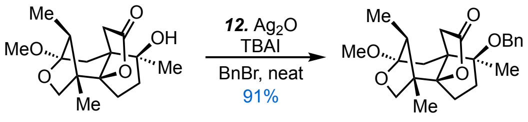


**33** **34**

Alcohol **33** (23 mg, 0.0796 mmol, 1.0 equiv), Ag$_2$O (184 mg, 0.796 mmol, 10.0 equiv), and TBAI (30 mg, 0.0796 mmol, 1.0 equiv) was added to a 10 mL flask, which was evacuated and backfilled with argon. Then BnBr (1 mL) was added and the reaction mixture was stirred at 20 °C for overnight. The reaction mixture was filtered through a silica gel pad and washed with EtOAc. The combined filtrate was concentrated under reduced pressure. The crude product was purified by flash chromatography using a gradient eluent system (hexanes to 4% to 33% EtOAc in hexanes) to afford the lactone **34** (28 mg, 0.0724 mmol, 91%) as a white solid.

**$[\alpha]_D^{21.2}$** = +15.6 ($c$ = 0.1, CHCl$_3$).

**IR (neat):** 2969, 2937, 2885, 1769, 1496, 1453, 1417, 1387, 1341, 1289, 1258, 1238, 1212, 1201, 1163, 1135, 1114, 1092, 1083, 1052 $cm^{-1}$.

**$^{1}$H NMR (400 MHz, $CDCl_3$):** δ 7.37 – 7.30 (m, 2H), 7.30 – 7.22 (m, 3H), 4.43 (s, 2H), 3.75 (d, $J$ = 9.7 Hz, 1H), 3.68 (d, $J$ = 9.7 Hz, 1H), 3.47 (d, $J$ = 18.7 Hz, 1H), 3.39 (s, 3H), 2.37 – 2.25 (m, 2H), 2.19 – 2.07 (m, 1H), 2.07 – 1.87 (m, 4H), 1.67 (ddd, $J$ = 13.0, 8.9, 7.3 Hz, 1H), 1.35 (s, 3H), 1.10 (s, 3H), 1.96 (d, $J$ = 6.9 Hz, 3H).

**$^{13}$C NMR (101 MHz, $CDCl_3$):** δ 175.9, 138.5, 128.6, 127.6, 127.0, 107.6, 100.8, 86.7, 77.4, 70.9, 64.4, 53.3, 50.8, 43.1, 43.0, 41.9, 33.5, 32.7, 17.4, 15.1, 8.5.

**HRMS *m/z* (APCI):** calc. for $C_{23}H_{31}O_5^+$ $[M+H]^+$: 387.2166, found: 387.2168.

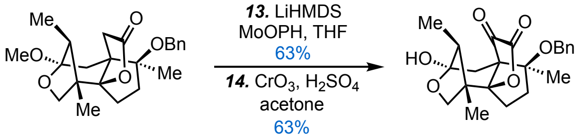


**34** **35**

To a solution of lactone **34** (28 mg, 0.0724 mmol, 1.0 equiv) in THF (3.5 mL) was added LiHMDS (435 mL, 1.0 M in THF, 0.435 mmol, 6.0 equiv) at –78 °C. The resultant mixture was stirred at –78 °C for 1 hour before MoOPH (63 mg, 0.145 mmol, 2.0 equiv) was added portionwise. The reaction mixture was allowed to warm to –20 °C and was then quenched with saturated aqueous $NaHCO_3$ and extracted with EtOAc. The combined organic layers were washed with brine, dried over $Na_2SO_4$ and concentrated under reduced pressure. The crude product was purified by flash chromatography using a gradient eluent system (4% to 10% to 33% EtOAc in hexanes) to afford α-hydroxylactone (18.3 mg, 0.0455 mmol, 63%) as a 3:1 mixture of diastereomers, which was used directly for the subsequent oxidation.

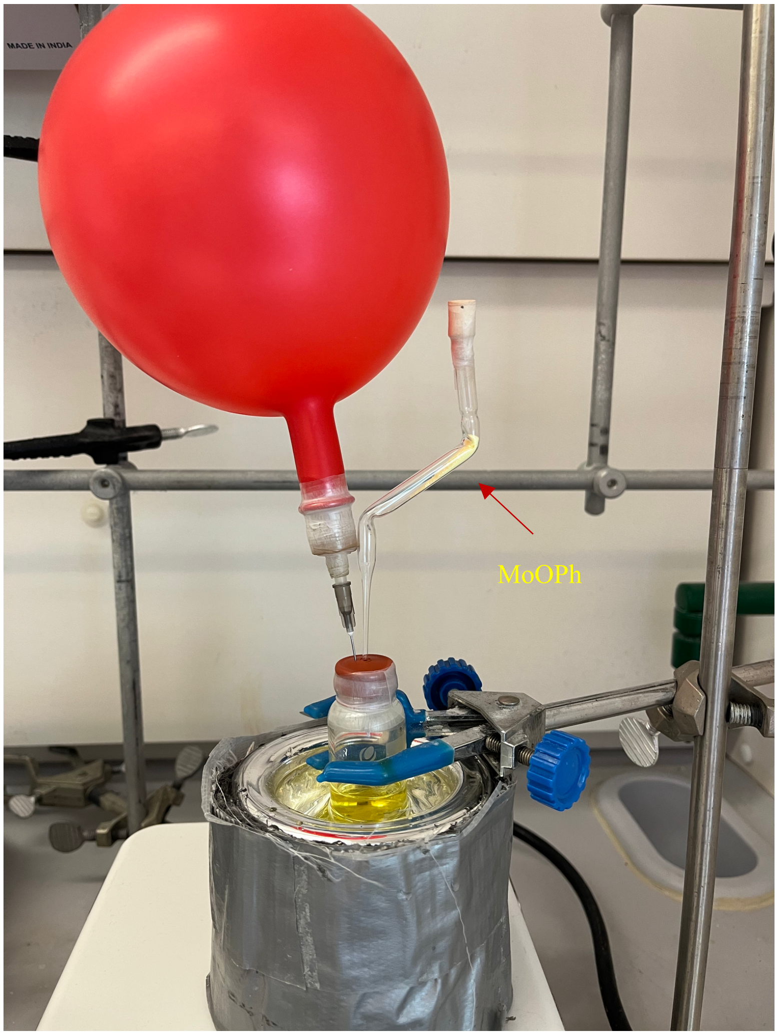


**Scheme S1.** Adding MoOPh portionwise under argon atmosphere. MoOPh was pre-stored in a bent Pasteur pipette linked to the vial in the glovebox. Tap the pipette to add MoOPh portionwise.

To a solution of the above α-hydroxylactone (7.8 mg, 0.019 mmol, 1.0 equiv) in acetone was added Jones reagent (38 μL, 2.5 M, 0.095 mmol, 5 equiv) at 0 °C. The resultant reaction was stirred at 0 °C for 30 min before it was quenched with *i*-PrOH. The reaction mixture was allowed to warm to room temperature and stirred for 10 min before aqueous 2 M HCl was added. The resultant mixture was stirred at 20 °C overnight. The crude product was subjected to flash column chromatography (eluent: 4% to 25% to 35% acetone in hexanes) to yield α-ketolactone **35** (4.7 mg, 0.012 mmol, 63%) as a yellow solid.

$[\alpha]_D^{21.2}$ = +12.4 (*c* = 0.2 , $CHCl_3$).

**IR (neat):** 3194, 2924, 2854, 1748, 1594, 1454, 1382, 1259, 1214, 1180, 1156, 1100, 1081, 1011 $cm^{-1}$.

**$^1$H NMR (800 MHz, $CDCl_3$):** δ 7.33 – 7.28 (t, *J* = 7.6 Hz, 2H), 7.25 – 7.22 (m, 1H), 7.15 – 7.10 (m, 2H), 4.39 (d, *J* = 11.6 Hz, 1H), 4.33 (d, *J* = 11.5 Hz, 1H), 3.76 (d, *J* = 9.8 Hz, 1H), 3.66 (d, *J* = 9.9 Hz, 1H), 2.78

(d, $J$ = 15.0 Hz, 1H), 2.60 (s, 1H), 2.41 – 2.31 (m, 3H), 2.09 (d, $J$ = 15.0 Hz, 1H), 1.69 – 1.61 (m, 1H), 1.39 (s, 3H), 1.18 (s, 3H), 1.08 (q, $J$ = 6.8 Hz, 1H), 0.94 (d, $J$ = 6.8 Hz, 3H).

**$^{13}$C NMR (201 MHz, CDCl$_3$):** δ 196.4, 159.3, 137.2, 128.6, 127.8, 126.9, 104.9, 100.3, 93.0, 70.1, 64.5, 57.5, 50.9, 42.6, 41.6, 34.8, 33.5, 16.7, 15.5, 7.9.

**HRMS *m*/*z* (APCI):** calc. for $C_{22}H_{27}O_6^+$ [M+H]$^+$: 387.1802, found: 387.1802.

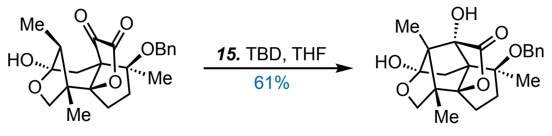


To a solution of α-ketolactone **35** (7.2 mg, 0.0186 mmol, 1.0 equiv) in THF (1 mL), was added a solution of TBD in $CH_2Cl_2$ (10.0 μL, 0.26 mg TBD, 1.86 μmol, 0.026 mg/μL, 0.1 equiv) at 0 °C. The resulting mixture was stirred at 0 °C for 7 min before it was quenched with aqueous 0.5 M HCl. The resulting mixture was extracted with EtOAc. The combined organic layers were washed with brine, dried over $Na_2SO_4$, and concentrated under reduced pressure. The crude product was subjected to flash chromatography (eluent: 4% to 10% to 25% acetone in hexanes) to yield lactone **36** (4.4 mg, 0.0114 mmol, 61%) as a white solid and recoverd α-ketolactone (2.5 mg, 0.00647 mmol, 35%).

**$[\alpha]_D^{21.8}$** = +6.60 ($c$ = 0.1 , CHCl$_3$).

**IR (neat):** 3374, 2923, 2854, 1775, 1661, 1457, 1378, 1307, 1243, 1202, 1146, 1122, 1092, 1042 cm$^{-1}$.

**$^1$H NMR (800 MHz, CDCl$_3$):** δ 7.37 – 7.29 (m, 2H), 7.28 – 7.26 (m, 2H), 7.26 – 7.23 (d, $J$ = 7.3 Hz, 1H), 4.47 (d, $J$ = 11.3 Hz, 1H), 4.40 (d, $J$ = 11.3 Hz, 1H), 3.74 (d, $J$ = 10.1 Hz, 1H), 3.63 (d, $J$ = 10.1 Hz, 1H), 2.89 (d, $J$ = 8.2 Hz, 1H), 2.80 (ddd, $J$ = 13.9, 10.3, 8.2 Hz, 1H), 2.75 (s, 1H), 2.47 (d, $J$ = 14.6 Hz, 1H), 2.03 (tdd, $J$ = 13.9, 8.6, 1.5 Hz, 2H), 1.98 (d, $J$ = 14.6 Hz, 1H), 1.88 (ddd, $J$ = 13.8, 10.3, 9.2 Hz, 1H), 1.35 (s, 3H), 1.10 (s, 3H), 0.96 (s, 3H).

**$^{13}$C NMR (151 MHz, CDCl$_3$):** δ 174.2, 138.3, 128.5, 127.6, 127.4, 109.9, 101.9, 87.4, 81.4, 69.5, 69.2, 65.5, 62.3, 52.0, 40.8, 38.1, 23.9, 23.0, 16.7, 5.2.

**HRMS *m*/*z* (APCI):** calc. for $C_{22}H_{25}O_6^-$ [M-H]$^-$: 385.1657, found: 385.1665.

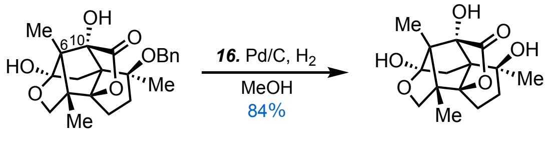


To a solution of the lactone **36** (4.4 mg, 0.0113 mmol, 1.0 equiv) in MeOH (1 mL) was added 10% Pd/C (2.5 mg, 1.13 μmol, 0.1 equiv, 50% wet with water for safety). The reaction mixture was stirred under a hydrogen atmosphere by bubbling $H_2$ gas through the solution for 10 min. Bubbling was then stopped, and

the reaction was allowed to stir under hydrogen atmosphere at room temperature for overnight. After completion, the reaction mixture was filtered through a silica gel pad, washed thoroughly with EtOAc and concentrated under reduced pressure. The resulting crude product was purified by flash chromatography (eluent: 2% to 8% to 20% MeOH in $CH_2Cl_2$) to afford illisimonin A (2.8 mg, 0.00945 mmol, 84%) as a white solid.

**$[\alpha]_D^{22.3}$** = -14.9 (*c* = 0.2, MeOH).

**IR (neat):** 3355, 2962, 2925, 2854, 1769, 1632, 1456, 1378, 1307, 1199, 1039, 1005 $cm^{-1}$.

**$^1$H NMR (600 MHz, Methanol-$d_4$):** δ 3.80 (d, *J* = 10.1 Hz, 1H), 3.56 (d, *J* = 10.1 Hz, 1H), 2.38 (dt, *J* = 14.2, 8.6 Hz, 1H), 2.32 (d, *J* = 14.3 Hz, 1H), 2.26 (ddd, *J* = 14.0, 10.4, 3.1 Hz, 1H), 2.03 (ddd, *J* = 14.4, 10.4, 8.4 Hz, 1H), 1.98 (d, *J* = 14.3 Hz, 1H), 1.92 (ddd, *J* = 14.4, 8.9, 3.1 Hz, 1H), 1.28 (s, 3H), 1.04 (s, 3H), 0.92 (s, 3H).

**$^{13}$C NMR (151 MHz, Methanol-$d_4$):** δ 177.6, 111.5, 104.9, 88.4, 76.7, 71.0, 69.7, 63.9, 52.8, 46.5, 39.1, 28.1, 23.1, 16.7, 5.9.

**HRMS *m/z* (APCI):** calc. for $C_{15}H_{19}O_6^-$ $[M-H]^-$: 295.1187, found: 295.1192.

## C. NMR Comparison Tables

### Table S1. $^1$H NMR ($CD_3OD$) Spectroscopic Comparison of Natural & Our Synthetic illisimonin A

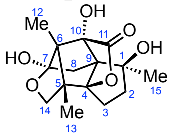


| No. | Natural, 500 MHz δ $^1$H [ppm, mult, *J*(Hz)] | Synthetic, 600 MHz δ $^1$H [ppm, mult, *J*(Hz)] |
|---|---|---|
| 1 | – | – |
| 2a | 2.25 (ddd, *J* = 14.0, 10.5, 3.5 Hz, 1H) | 2.26 (ddd, *J* = 14.0, 10.4, 3.1 Hz, 1H) |
| 2b | 2.36 (d app. t, *J* = 14.0, 8.5 Hz, 1H) | 2.38 (d app. t, *J* = 14.2, 8.7 Hz, 1H) |
| 3a | 2.03 (ddd, *J* = 14.5, 10.5, 8.5 Hz, 1H) | 2.03 (ddd, *J* = 14.4, 10.4, 8.4 Hz, 1H) |
| 3b | 1.91 (ddd, *J* = 14.5, 8.5, 3.5 Hz, 1H) | 1.92 (ddd, *J* = 14.4, 8.9, 3.1 Hz, 1H) |
| 4 | – | – |
| 5 | – | – |
| 6 | – | – |
| 7 | – | – |
| 8a | 1.97 (d, *J* = 14.5 Hz, 1H) | 1.98 (d, *J* = 14.3 Hz, 1H) |
| 8b | 2.30 (d, *J* = 14.5 Hz, 1H) | 2.32 (d, *J* = 14.3 Hz, 1H) |
| 9 | – | – |
| 10 | – | – |
| 11 | – | – |
| 12 | 0.91 (s, 3H) | 0.92 (m, 3H) |
| 13 | 1.03 (s, 3H) | 1.04 (m, 3H) |
| 14a | 3.79 (d, *J* = 10.0 Hz, 1H) | 3.80 (d, *J* = 10.1 Hz, 1H) |
| 14b | 3.55 (d, *J* = 10.0 Hz, 1H) | 3.56 (d, *J* = 10.1 Hz, 1H) |
| 15 | 1.27 (s, 3H) | 1.28 (s, 3H) |

**Table S2. $^1$H NMR ($CD_3OD$) Spectroscopic Comparison of Rychonovsky's, Kalesse's, Yang's and ours syntheses of illisimonin A**

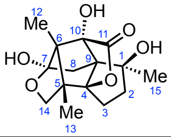


| No. | Rychonovsky's, 500 MHz δ $^1$H [ppm, mult, $J$(Hz)] | Kalesse's, 500 MHz δ $^1$H [ppm, mult, $J$(Hz)] | Yang's, 500 MHz δ $^1$H [ppm, mult, $J$(Hz)] | Ours, 600 MHz δ $^1$H [ppm, mult, $J$(Hz)] |
|---|---|---|---|---|
| 1 | – | – | – | – |
| 2a | 2.25 (ddd, $J$ = 14.0, 10.6, 3.3 Hz, 1H) | 2.25 (ddd, $J$ = 14.0, 10.4, 3.0 Hz, 1H) | 2.25 (ddd, $J$ = 14.0, 10.5, 3.5 Hz, 1H) | 2.26 (ddd, $J$ = 14.0, 10.4, 3.1 Hz, 1H) |
| 2b | 2.37 (d app. t, $J$ = 14.2, 8.7 Hz, 1H) | 2.36 (ddd, $J$ = 14.2, 8.7, 8.7 Hz, 1H) | 2.36 (ddd, $J$ = 14.0, 8.5, 8.5 Hz, 1H) | 2.38 (d app. t, $J$ = 14.2, 8.7 Hz, 1H) |
| 3a | 2.02 (ddd, $J$ = 14.3, 10.3, 8.5 Hz, 1H) | 2.05-2.01 (m, 1H) | 2.03 (ddd, $J$ = 14.5, 10.5, 8.5 Hz, 1H) | 2.03 (ddd, $J$ = 14.4, 10.4, 8.4 Hz, 1H) |
| 3b | 1.91 (ddd, $J$ = 14.3, 8.9, 3.1 Hz, 1H) | 1.91 (ddd, $J$ = 14.3, 8.9, 3.0 Hz, 1H) | 1.91 (ddd, $J$ = 14.5, 8.5, 3.5 Hz, 1H) | 1.92 (ddd, $J$ = 14.4, 8.9, 3.1 Hz, 1H) |
| 4 | – | – | – | – |
| 5 | – | – | – | – |
| 6 | – | – | – | – |
| 7 | – | – | – | – |
| 8a | 1.97 (d, J = 14.3 Hz, 1H) | 1.98 (d, $J$ = 14.3 Hz, 1H) | 1.97 (d, $J$ = 14.5 Hz, 1H) | 1.98 (d, $J$ = 14.3 Hz, 1H) |
| 8b | 2.31 (d, $J$ = 14.3 Hz, 1H) | 2.31 (d, $J$ = 14.3 Hz, 1H) | 2.30 (d, $J$ = 14.5 Hz, 1H) | 2.32 (d, $J$ = 14.3 Hz, 1H) |
| 9 | – | – | – | – |
| 10 | – | – | – | – |
| 11 | – | – | – | – |
| 12 | 0.91 (s, 3H) | 0.91 (s, 3H) | 0.91 (s, 3H) | 0.92 (m, 3H) |
| 13 | 1.03 (s, 3H) | 1.03 (s, 3H) | 1.03 (s, 3H) | 1.04 (m, 3H) |
| 14a | 3.79 (d, $J$ = 10.1 Hz, 1H) | 3.79 (d, $J$ = 10.1 Hz, 1H) | 3.79 (d, $J$ = 10.0 Hz, 1H) | 3.80 (d, $J$ = 10.1 Hz, 1H) |
| 14b | 3.55 (d, $J$ = 10.1 Hz, 1H) | 3.55 (d, $J$ = 10.1 Hz, 1H) | 3.55 (d, $J$ = 10.0 Hz, 1H) | 3.56 (d, $J$ = 10.1 Hz, 1H) |
| 15 | 1.27 (s, 3H) | 1.27 (s, 3H) | 1.27 (s, 3H) | 1.28 (s, 3H) |

**Table S3. $^{13}C$ NMR ($CD_3OD$) Spectroscopic Comparison of Natural & Synthetic illisimonin A**

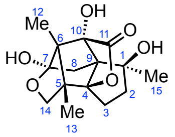


| No. | Natural, 126 MHz δ $^{13}C$ (ppm) | Synthetic, 151 MHz δ $^{13}C$ (ppm) |
|---|---|---|
| 1 | 76.7 | 76.7 |
| 2 | 46.5 | 46.5 |
| 3 | 23.1 | 23.1 |
| 4 | 104.9 | 104.9 |
| 5 | 52.8 | 52.8 |
| 6 | 63.9 | 63.9 |
| 7 | 111.5 | 111.5 |
| 8 | 39.0 | 39.1 |
| 9 | 71.0 | 71.0 |
| 10 | 88.4 | 88.4 |
| 11 | 177.6 | 177.6 |
| 12 | 6.0 | 5.9 |
| 13 | 16.7 | 16.7 |
| 14 | 69.7 | 69.7 |
| 15 | 28.1 | 28.1 |

**Table S4. $^{13}$C NMR ($CD_3OD$) Spectroscopic Comparison of Rychonovsky's, Kalesse's, Yang's and ours syntheses of illisimonin A**

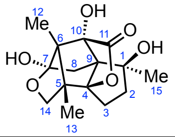

| No. | Rychonovsky's, 500 MHz δ $^{1}$H [ppm, mult, *J*(Hz)] | Kalesse's, 500 MHz δ $^{1}$H [ppm, mult, *J*(Hz)] | Yang's, 500 MHz δ $^{1}$H [ppm, mult, *J*(Hz)] | Ours, 600 MHz δ $^{1}$H [ppm, mult, *J*(Hz)] |
|---|---|---|---|---|
| 1 | 76.7 | 76.7 | 76.7 | 76.7 |
| 2 | 46.5 | 46.5 | 46.5 | 46.5 |
| 3 | 23.1 | 23.1 | 23.1 | 23.1 |
| 4 | 104.9 | 104.9 | 104.9 | 104.9 |
| 5 | 52.8 | 52.8 | 52.8 | 52.8 |
| 6 | 63.9 | 63.9 | 63.9 | 63.9 |
| 7 | 111.5 | 111.5 | 111.5 | 111.5 |
| 8 | 39.1 | 39.1 | 39.1 | 39.1 |
| 9 | 71.0 | 71.0 | 71.0 | 71.0 |
| 10 | 88.4 | 88.4 | 88.4 | 88.4 |
| 11 | 177.6 | 177.6 | 177.6 | 177.6 |
| 12 | 5.9 | 5.9 | 5.9 | 5.9 |
| 13 | 16.7 | 16.7 | 16.7 | 16.7 |
| 14 | 69.7 | 69.7 | 69.7 | 69.7 |
| 15 | 28.1 | 28.1 | 28.1 | 28.1 |

D. $^{1}$H and $^{13}$C NMR spectra

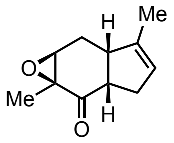


**25**
**(600 MHz, $CDCl_3$)**

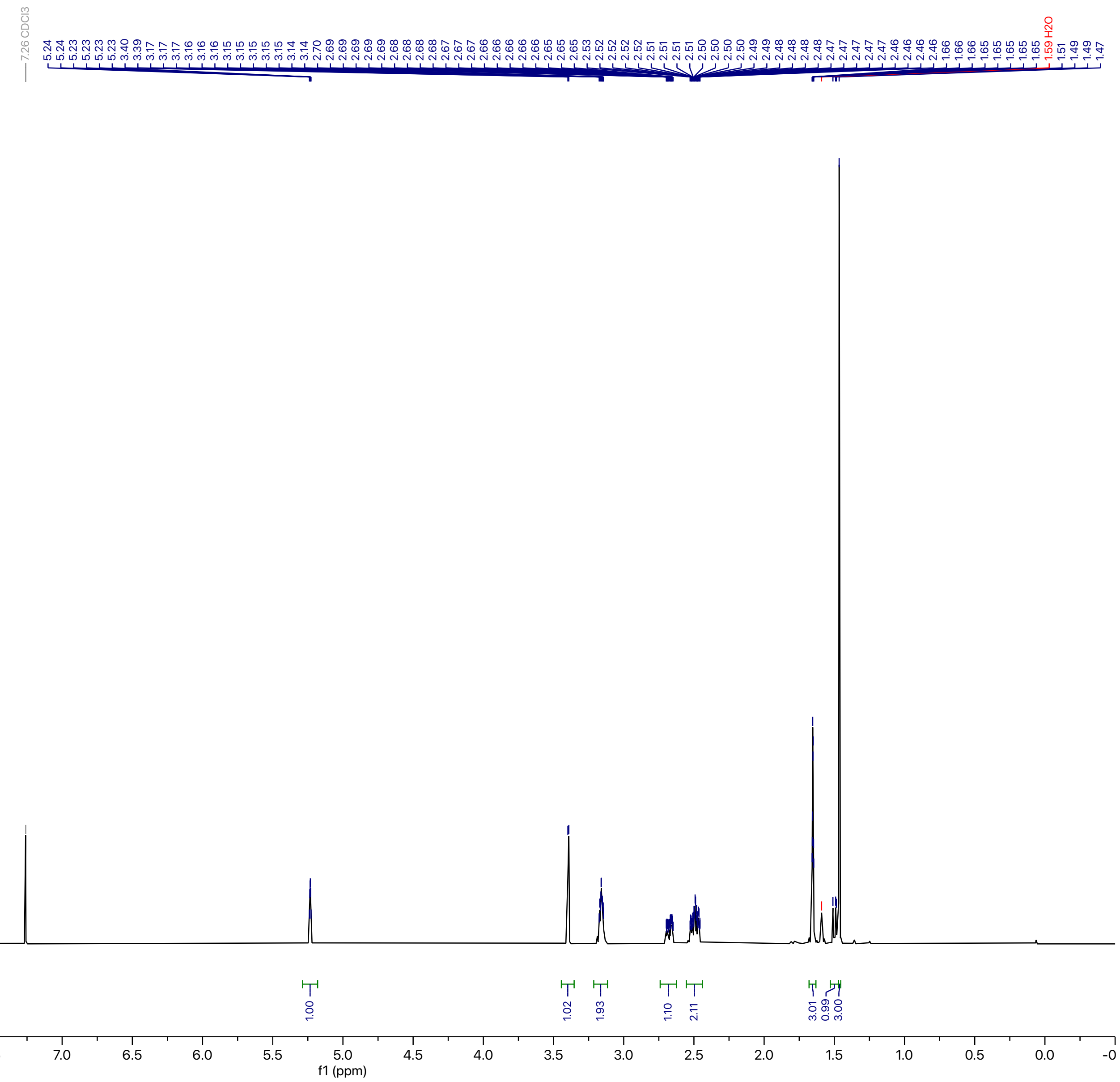

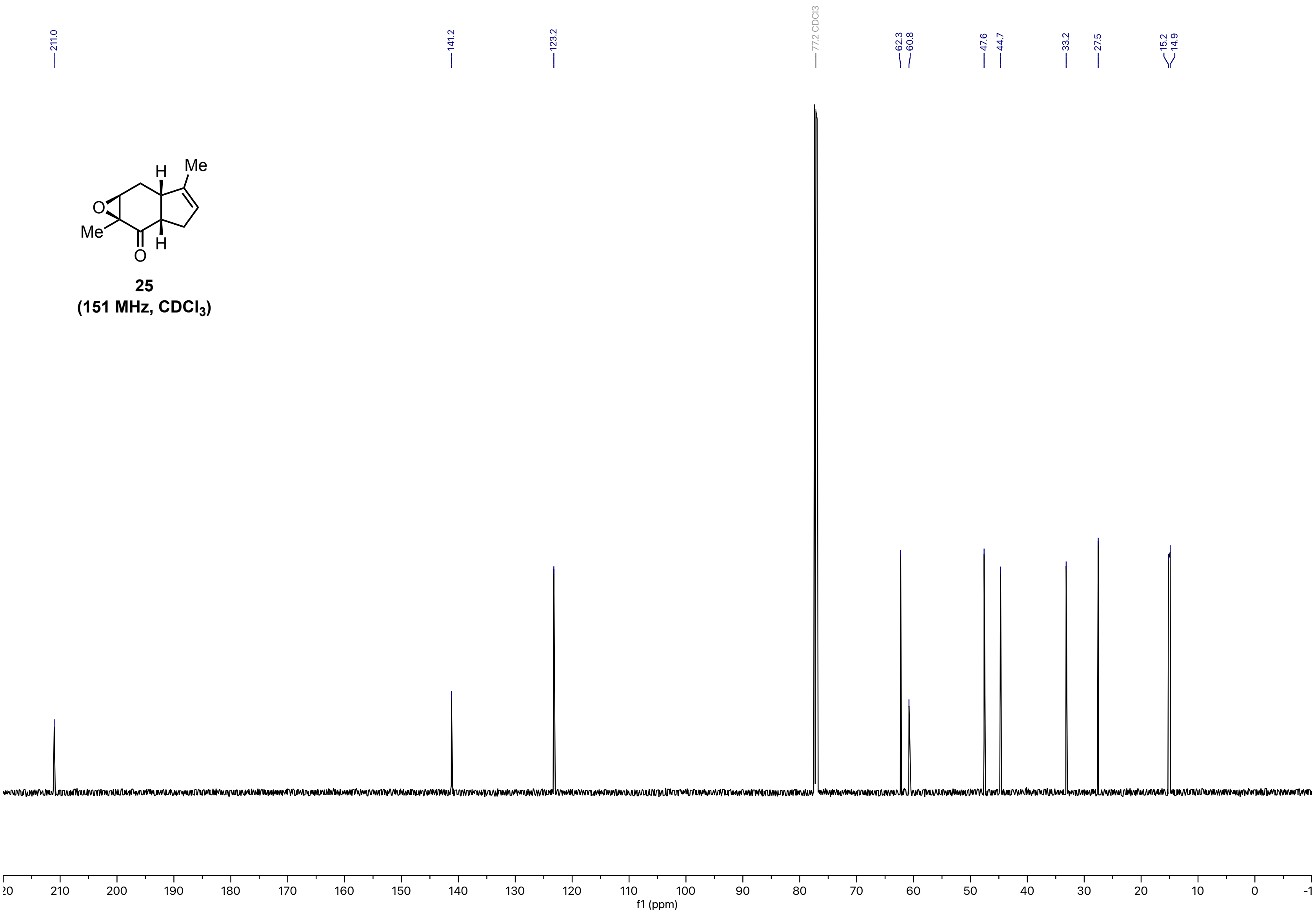
211.0
141.2
123.2
77.2 CDCl3
62.3
60.8
47.6
44.7
33.2
27.5
15.2
14.9
Me
H
O
Me
H
O
25
(151 MHz, CDCl3)
f1 (ppm)

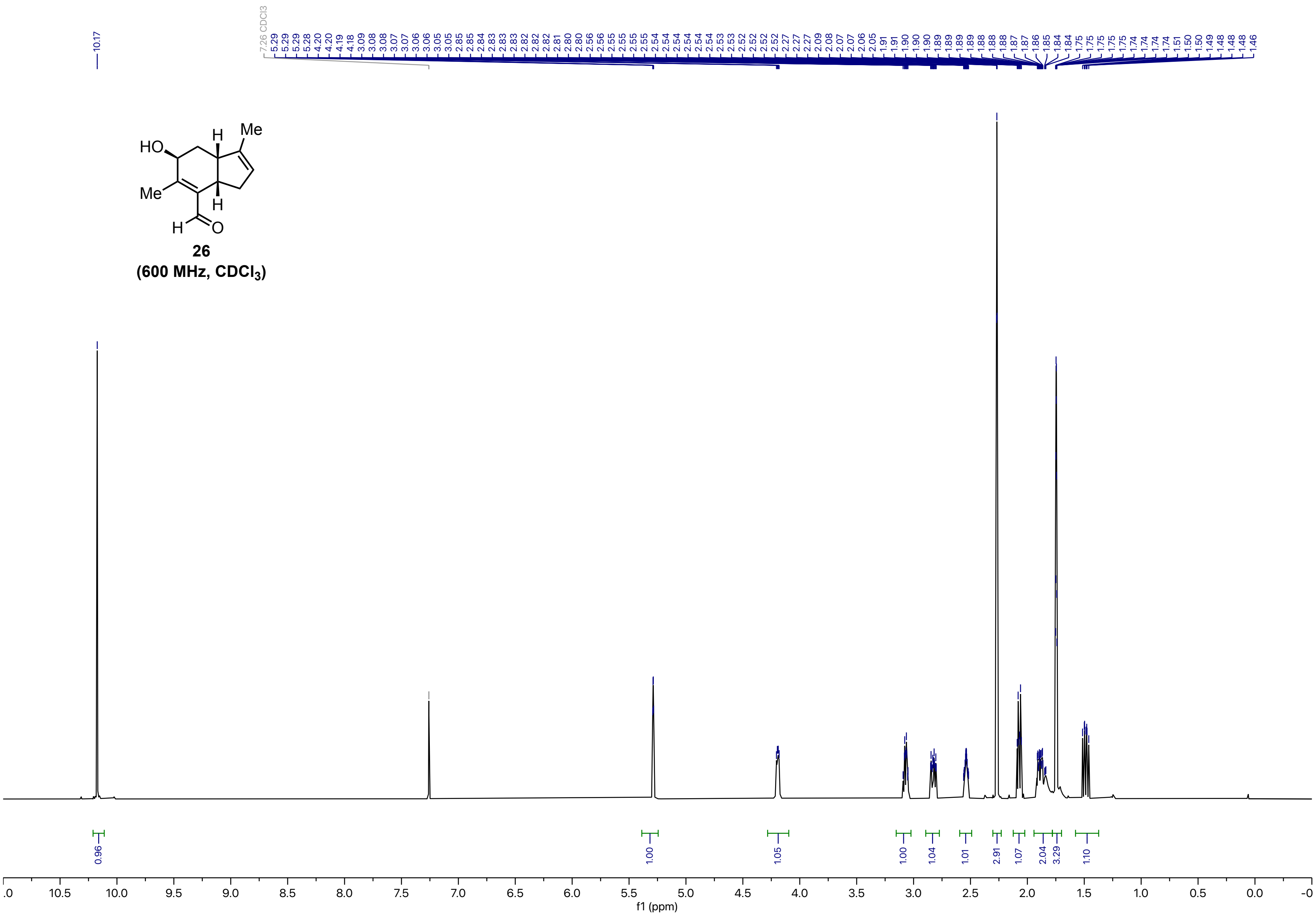
HO
Me
H
H
Me
H
O
26
(600 MHz, CDCl3)
f1 (ppm)

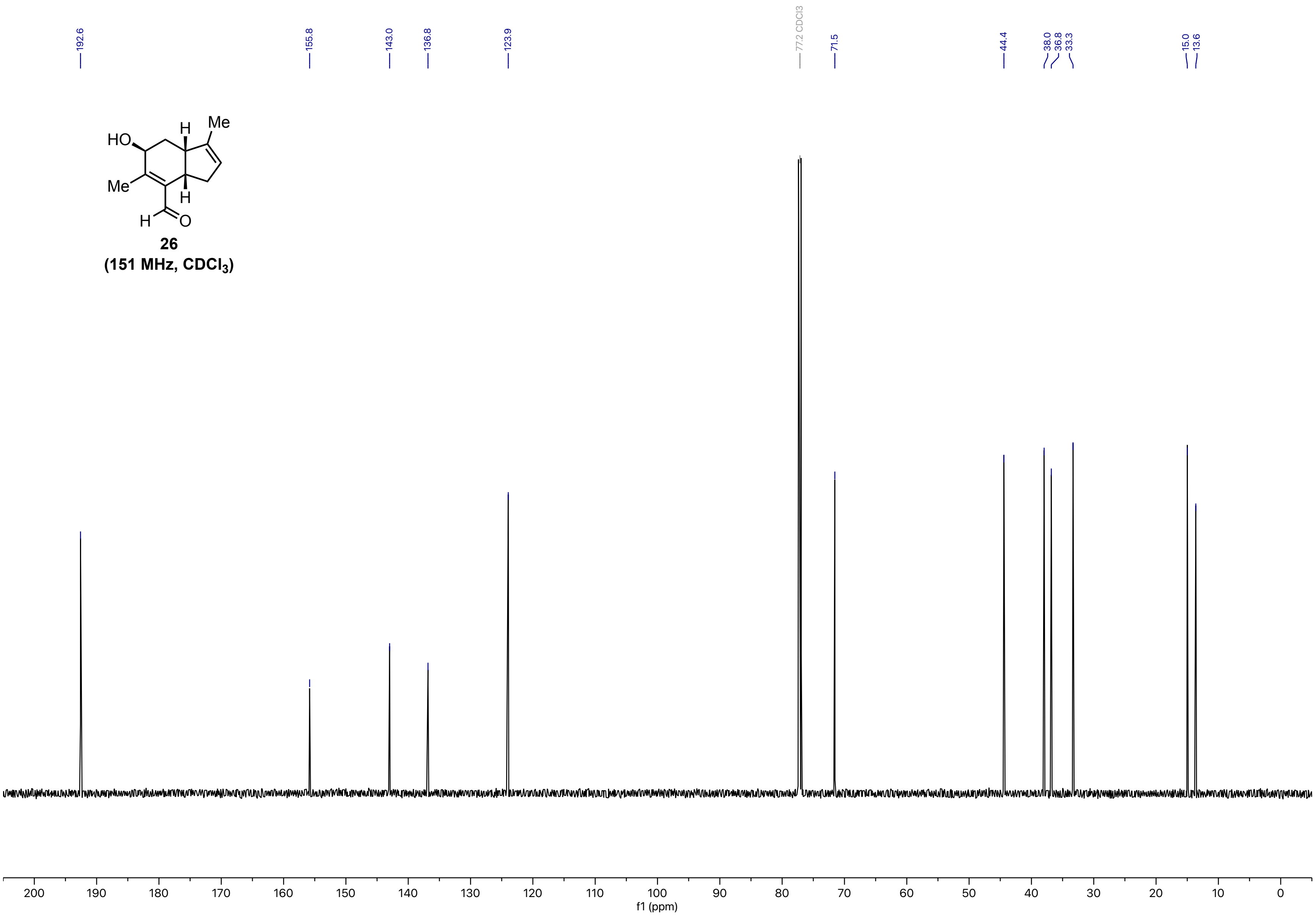

192.6
155.8
143.0
136.8
123.9
77.2 CDCl3
71.5
44.4
38.0
36.8
33.3
15.0
13.6
HO
H
Me
Me
H
H
O
26
(151 MHz, CDCl3)
200
190
180
170
160
150
140
130
120
110
100
90
80
70
60
50
40
30
20
10
0
f1 (ppm)

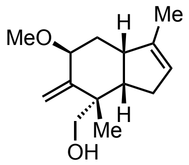


**27**

**(600 MHz, $CDCl_3$)**

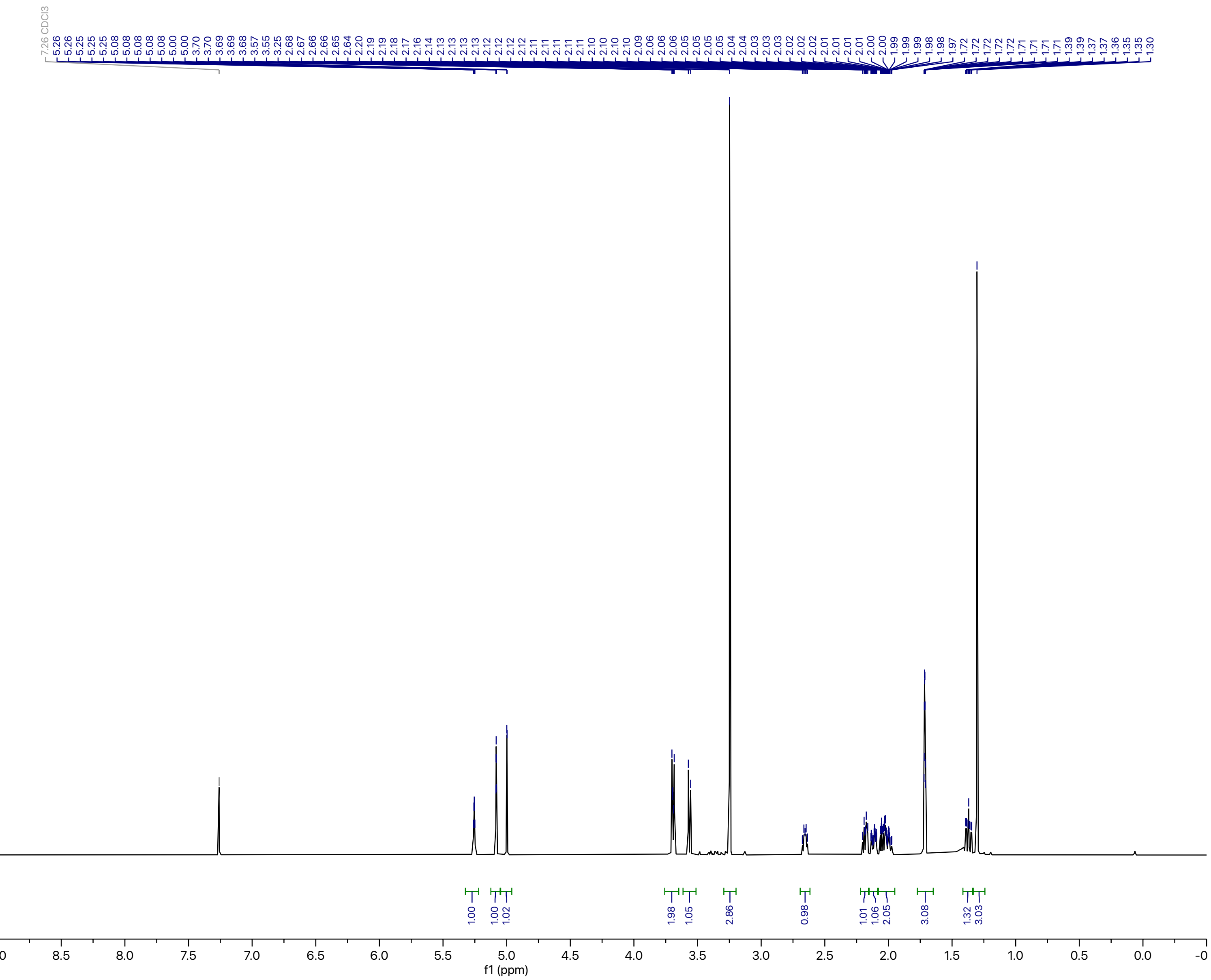

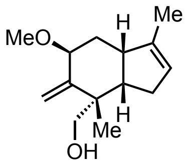


**27**

**(151 MHz, $CDCl_3$)**

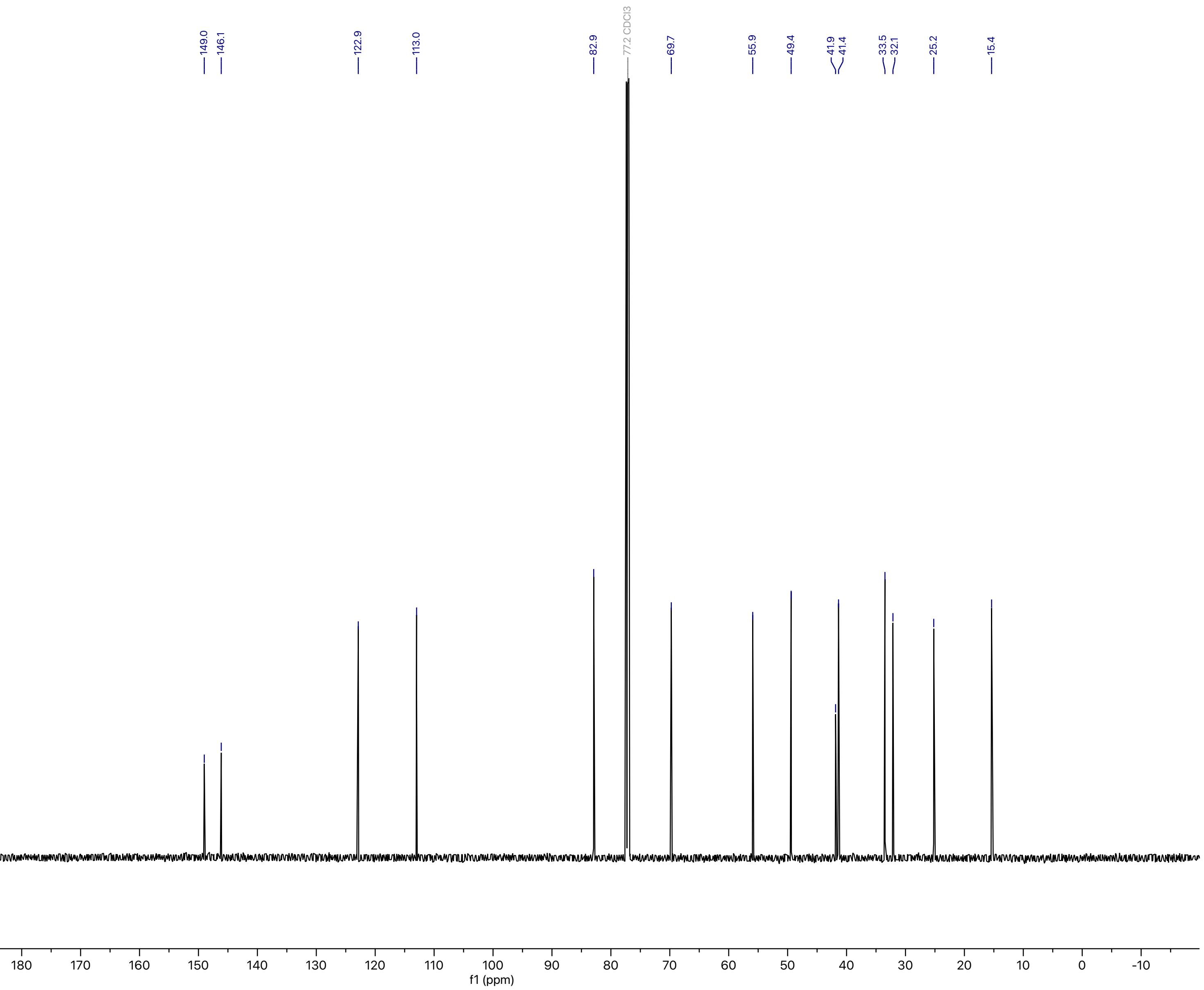

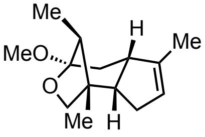


**28**
**(600 MHz, CDCl$_3$)**

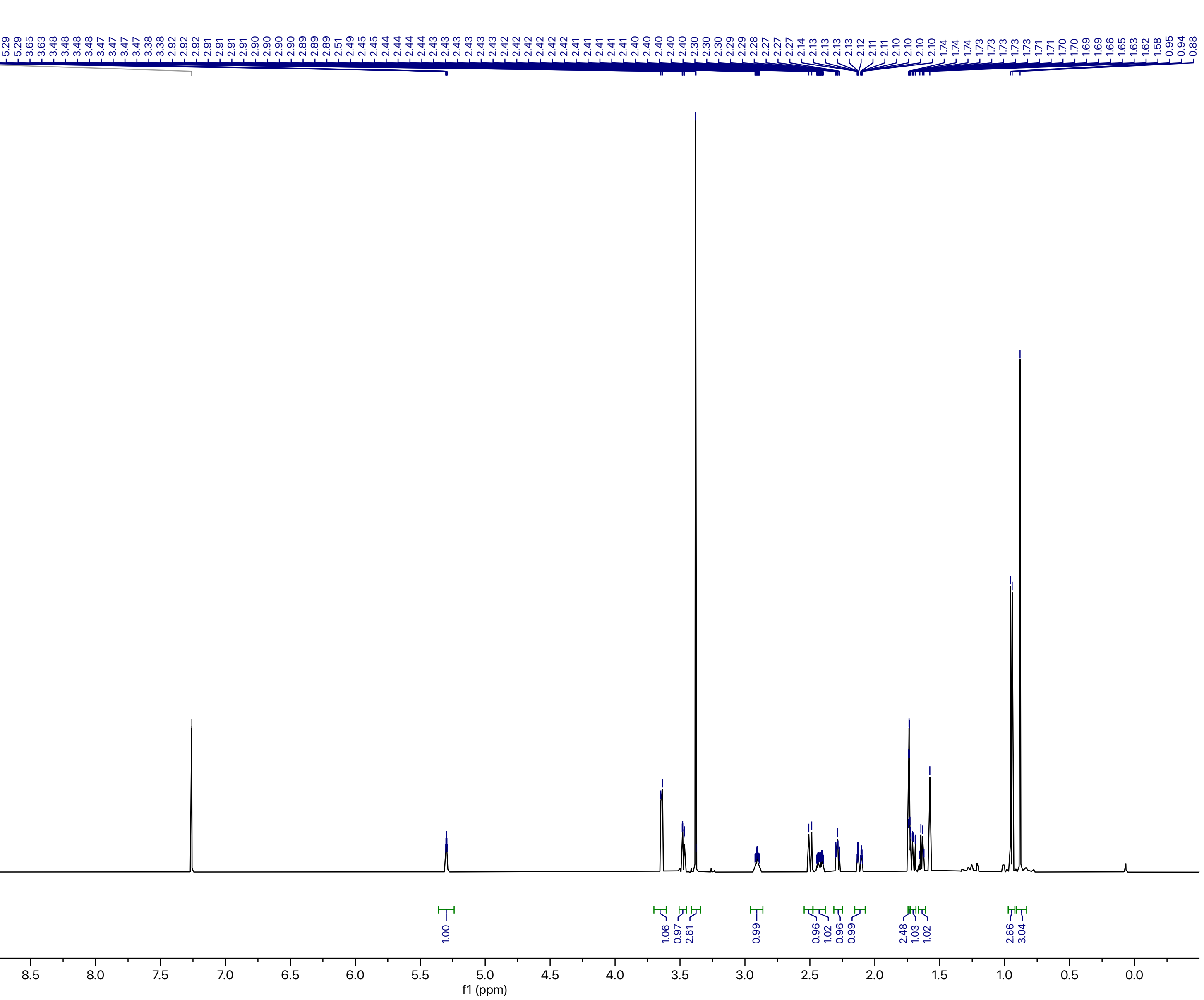

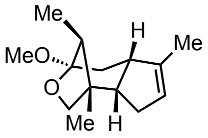


**28**
**(151 MHz, $CDCl_3$)**

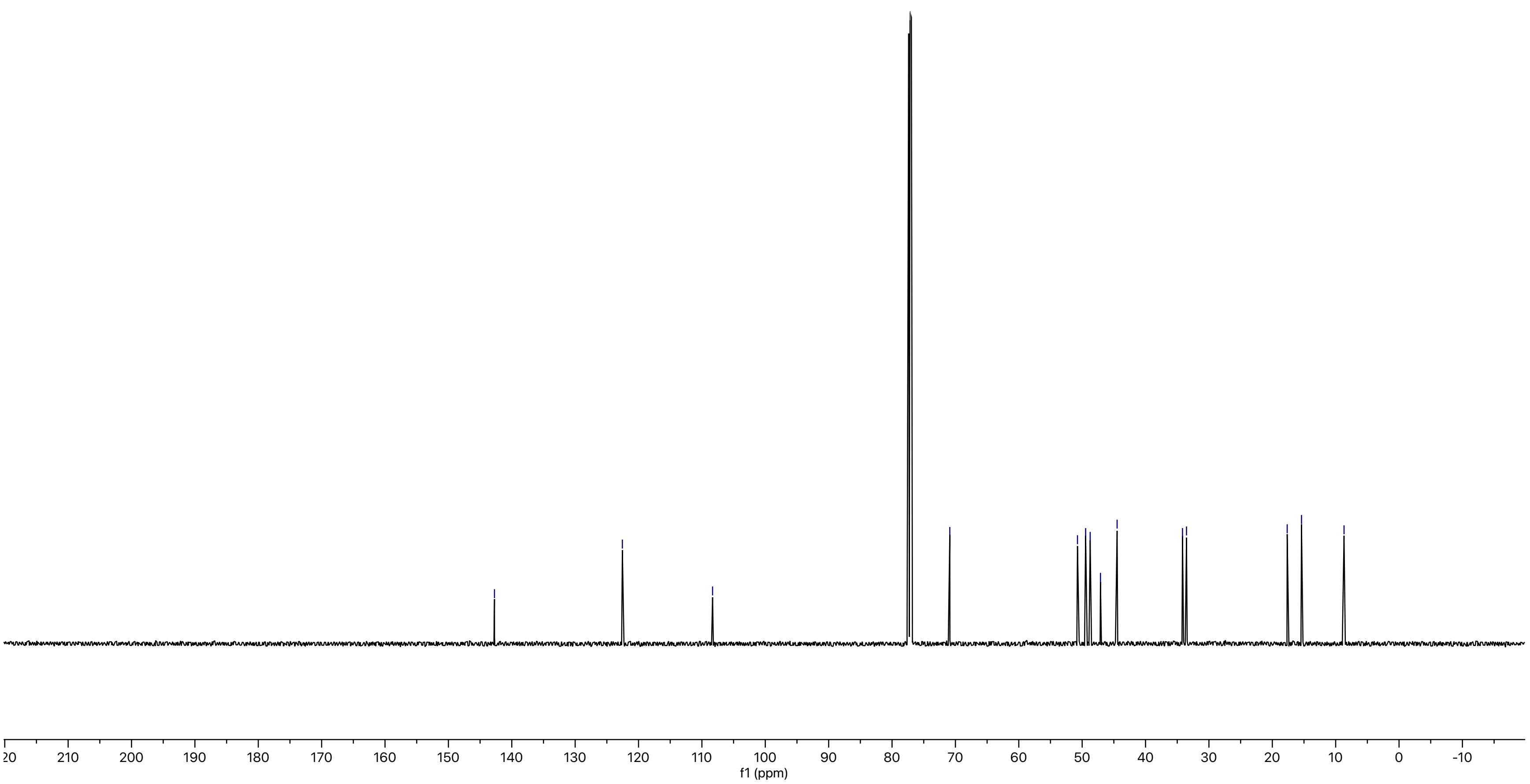

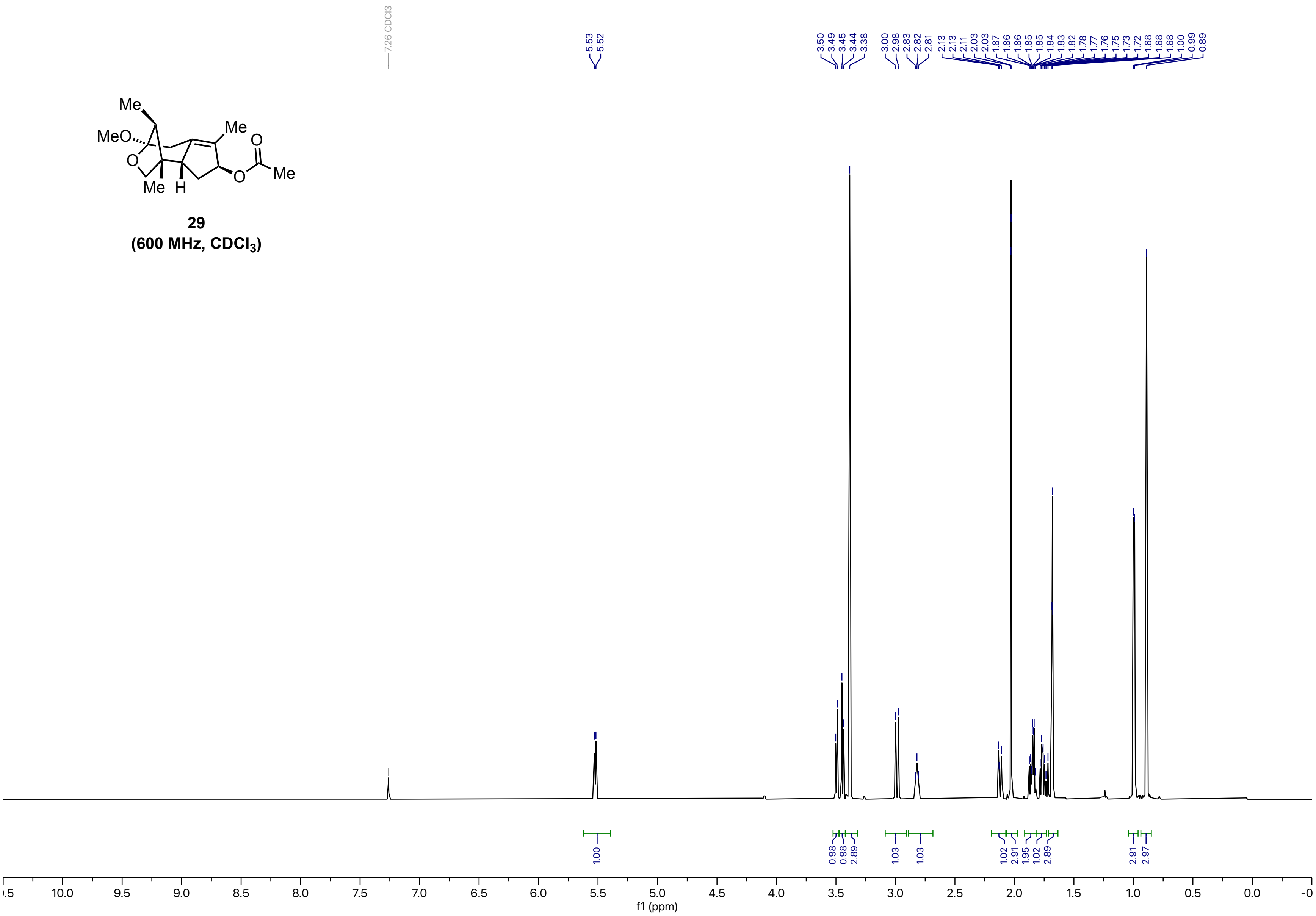
Me
MeO
O
Me
H
Me
O
O
Me
29
(600 MHz, CDCl3)
7.26 CDCl3
f1 (ppm)

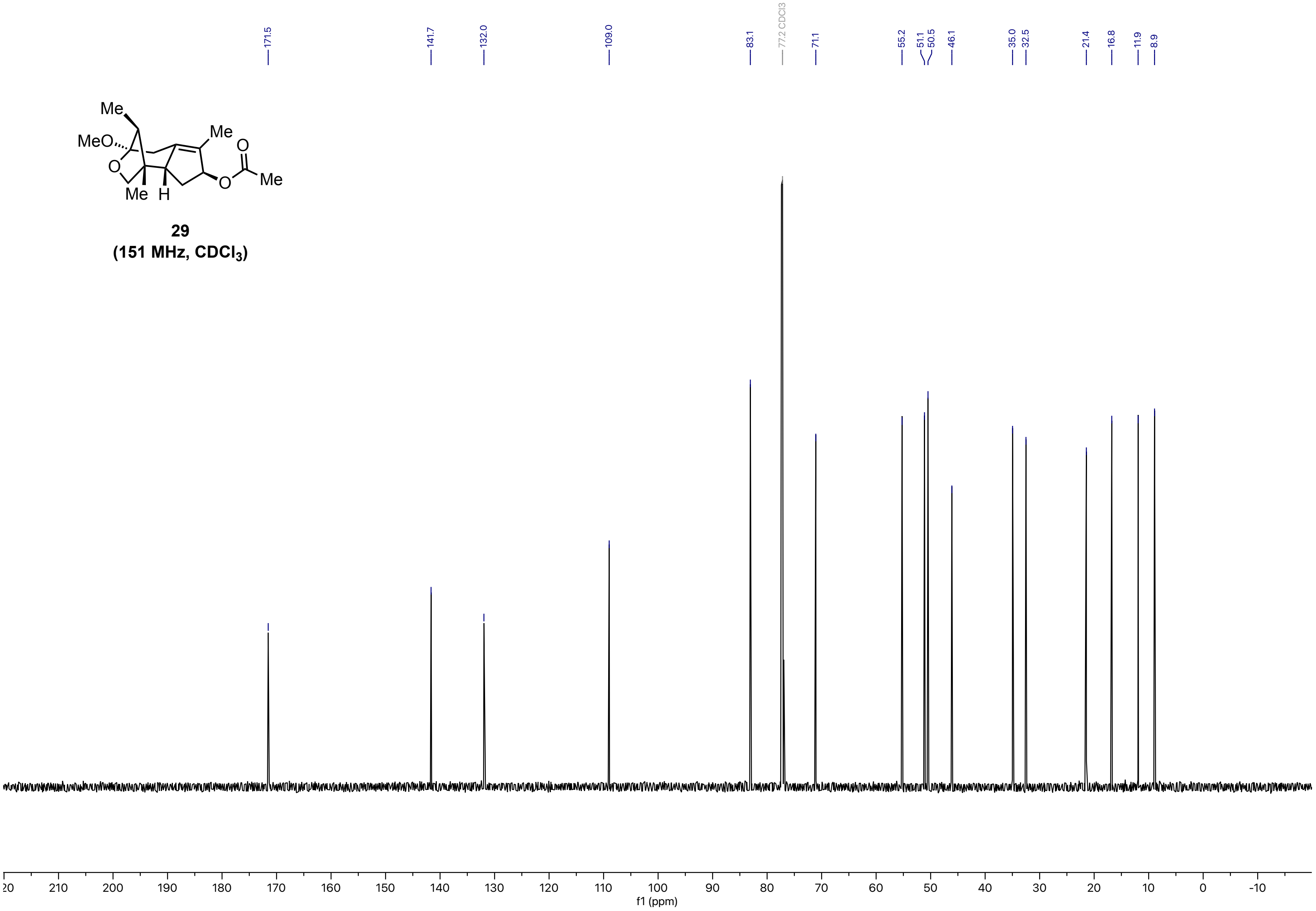


**29**
**(151 MHz, $CDCl_3$)**

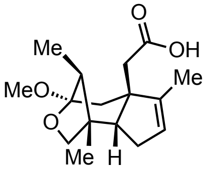


**30**
**(600 MHz, $CDCl_3$)**

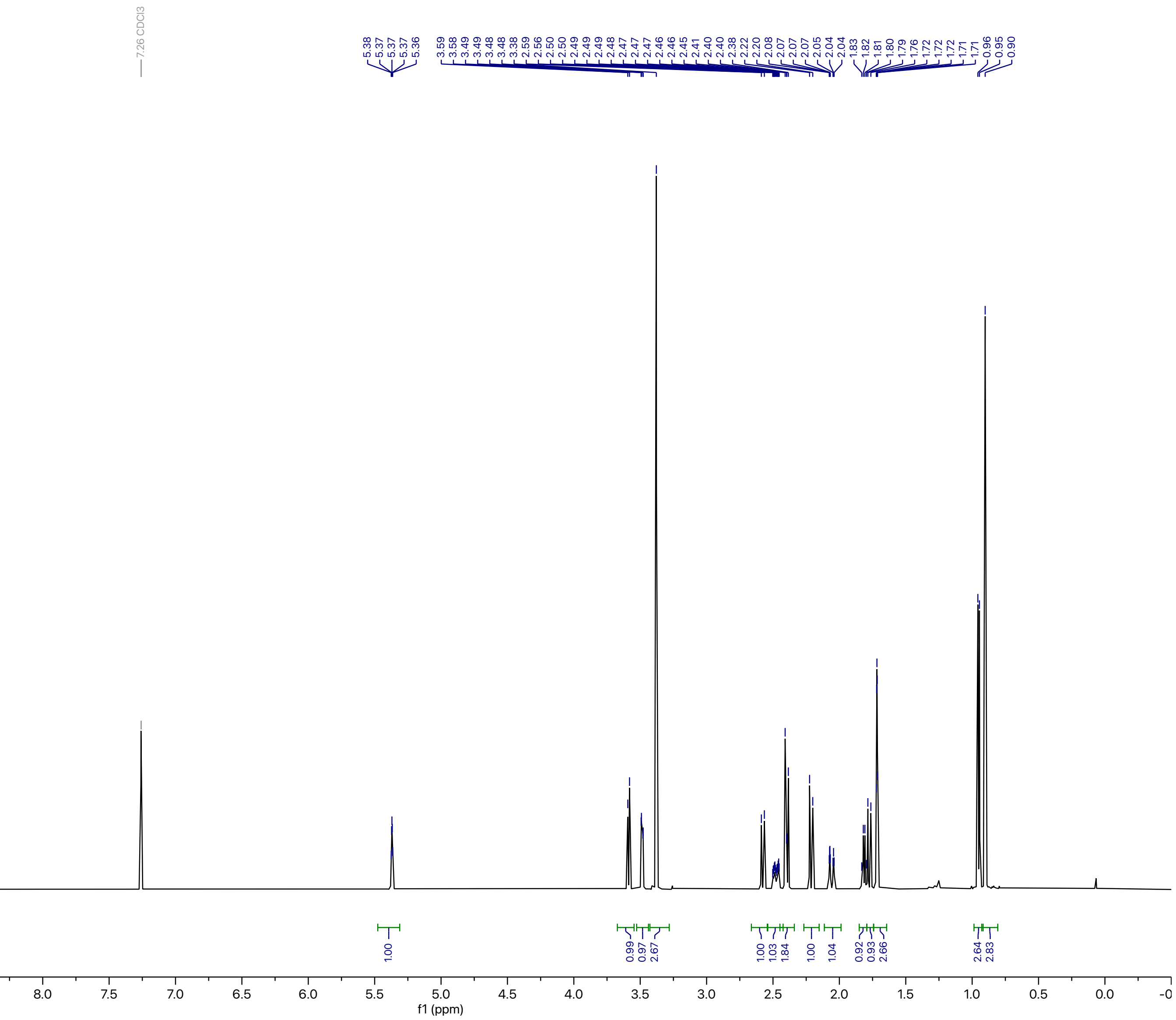

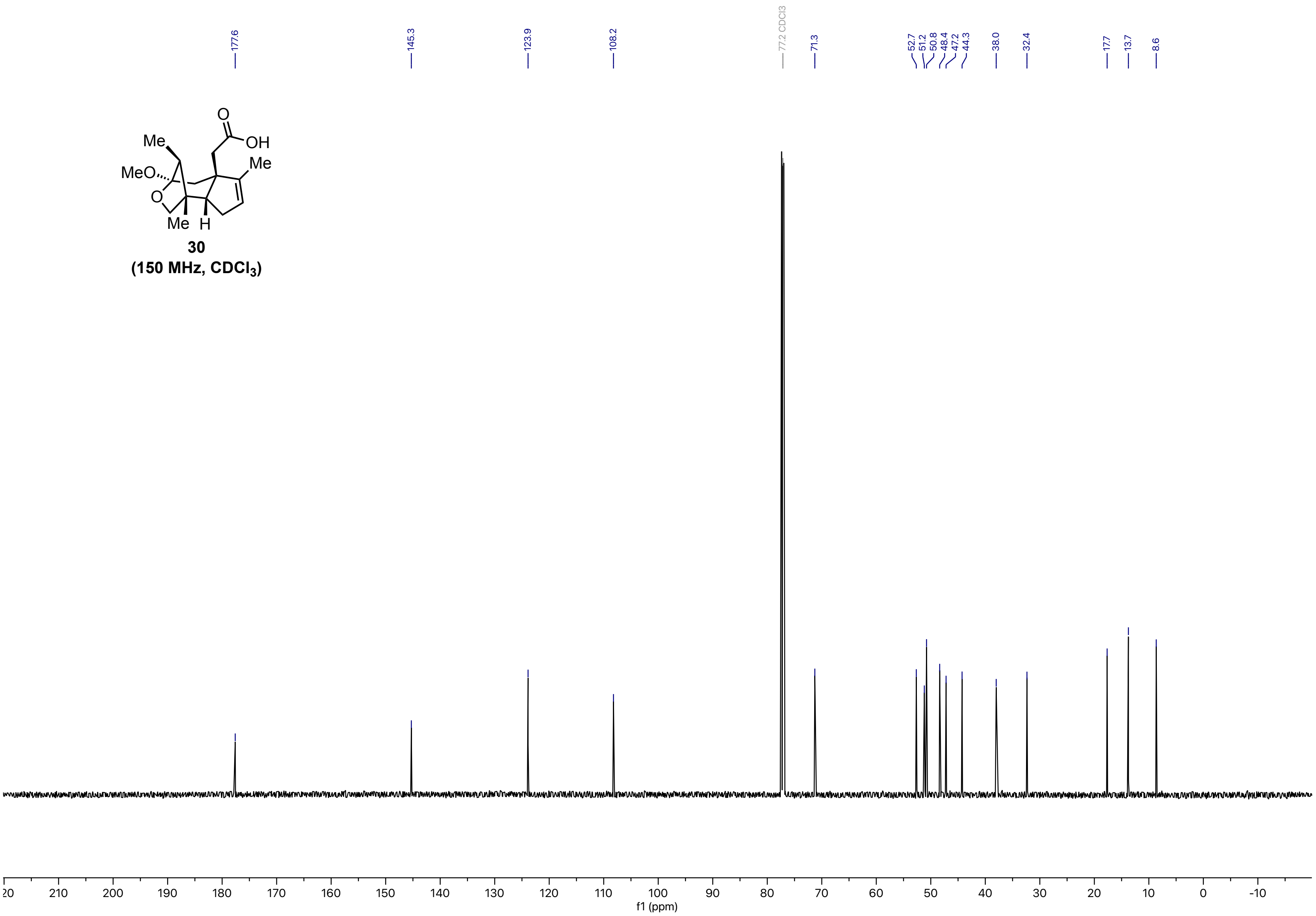

177.6
145.3
123.9
108.2
77.2 CDCl3
71.3
52.7
51.2
50.8
48.4
47.2
44.3
38.0
32.4
17.7
13.7
8.6
Me
MeO
O
Me
H
O
OH
Me
30
(150 MHz, CDCl3)
20
210
200
190
180
170
160
150
140
130
120
110
100
90
80
70
60
50
40
30
20
10
0
-10
f1 (ppm)

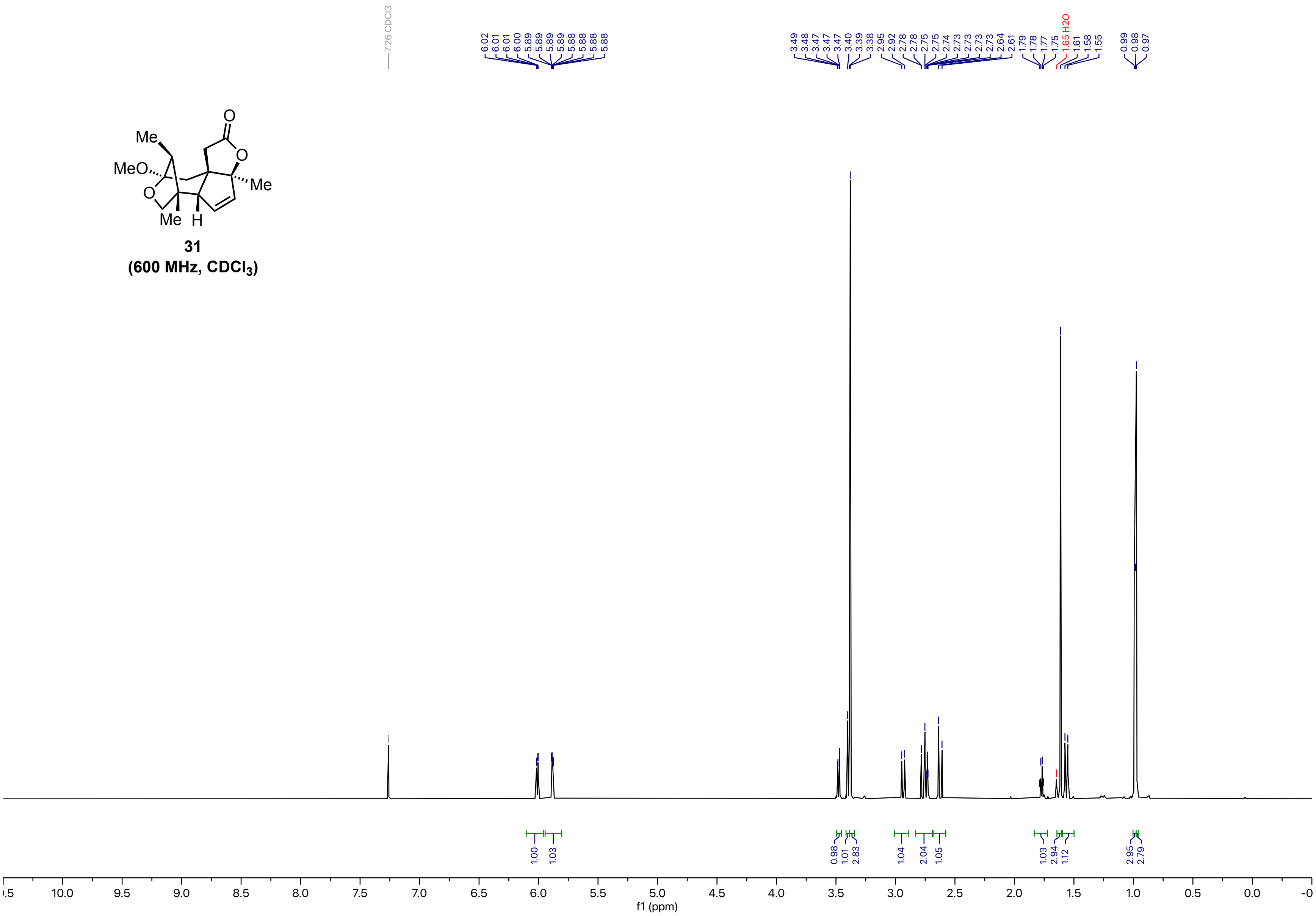


**31**
**(600 MHz, $CDCl_3$)**

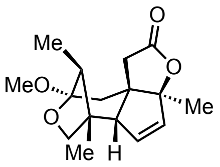

**31**
**(151 MHz, $CDCl_3$)**

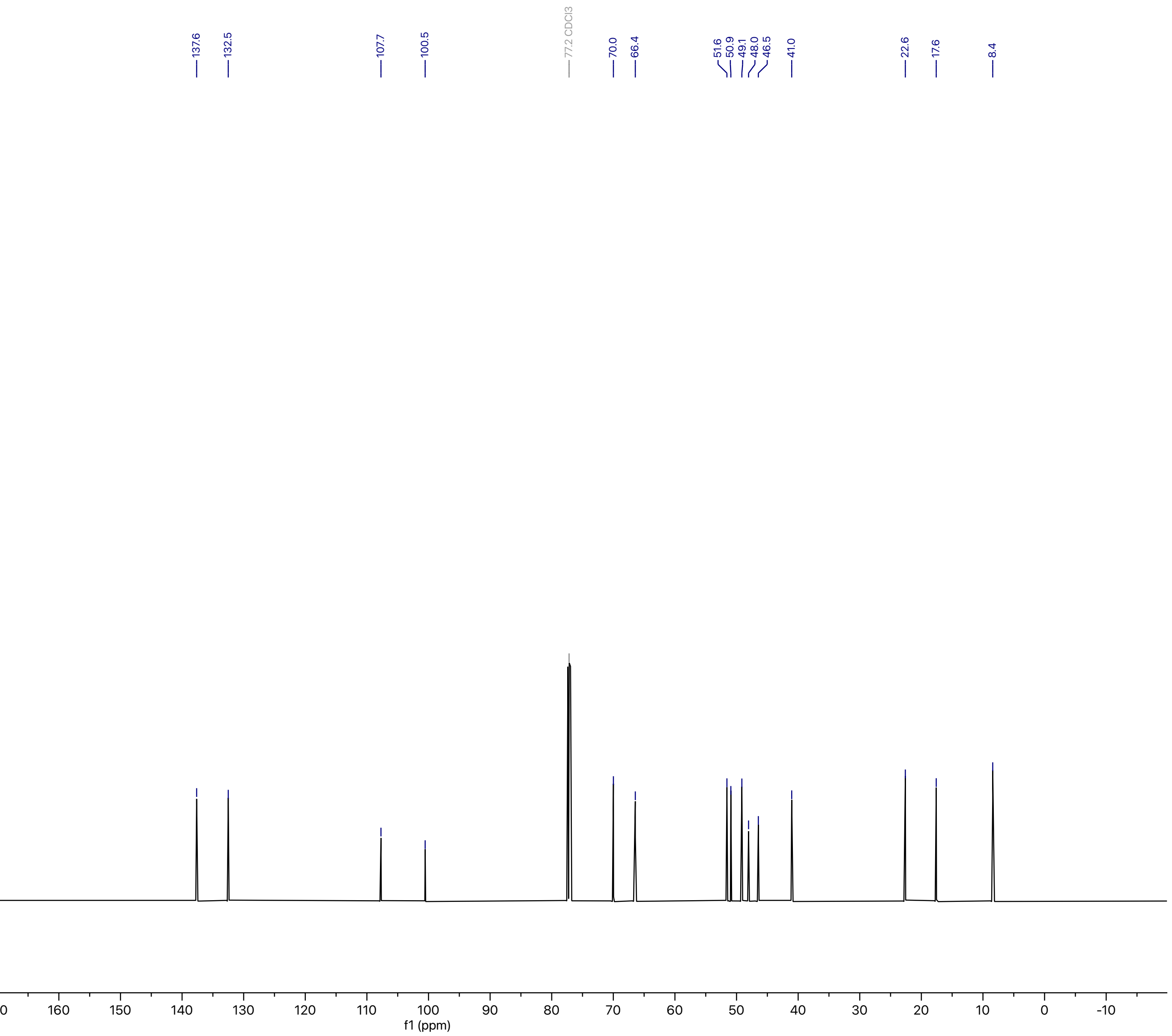

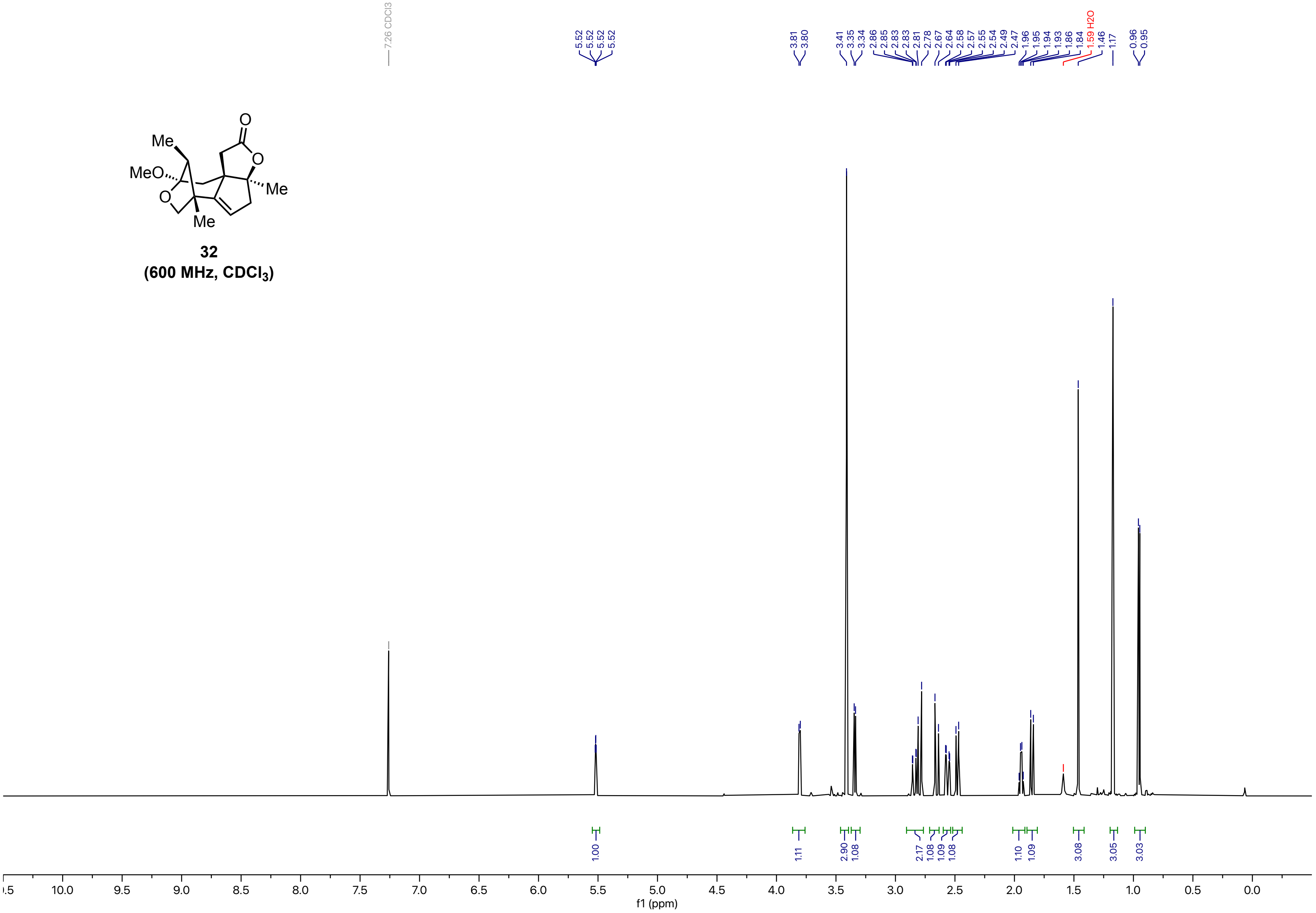
7.26 CDCl3
5.52
5.52
5.52
5.52
3.81
3.80
3.41
3.35
3.34
2.86
2.85
2.83
2.83
2.81
2.78
2.67
2.64
2.58
2.57
2.55
2.54
2.49
2.47
1.96
1.95
1.94
1.93
1.86
1.84
1.59 H2O
1.46
1.17
0.96
0.95
Me
MeO
O
O
O
Me
Me
32
(600 MHz, CDCl3)
1.00
1.11
2.90
1.08
2.17
1.08
1.09
1.08
1.10
1.09
3.08
3.05
3.03
f1 (ppm)

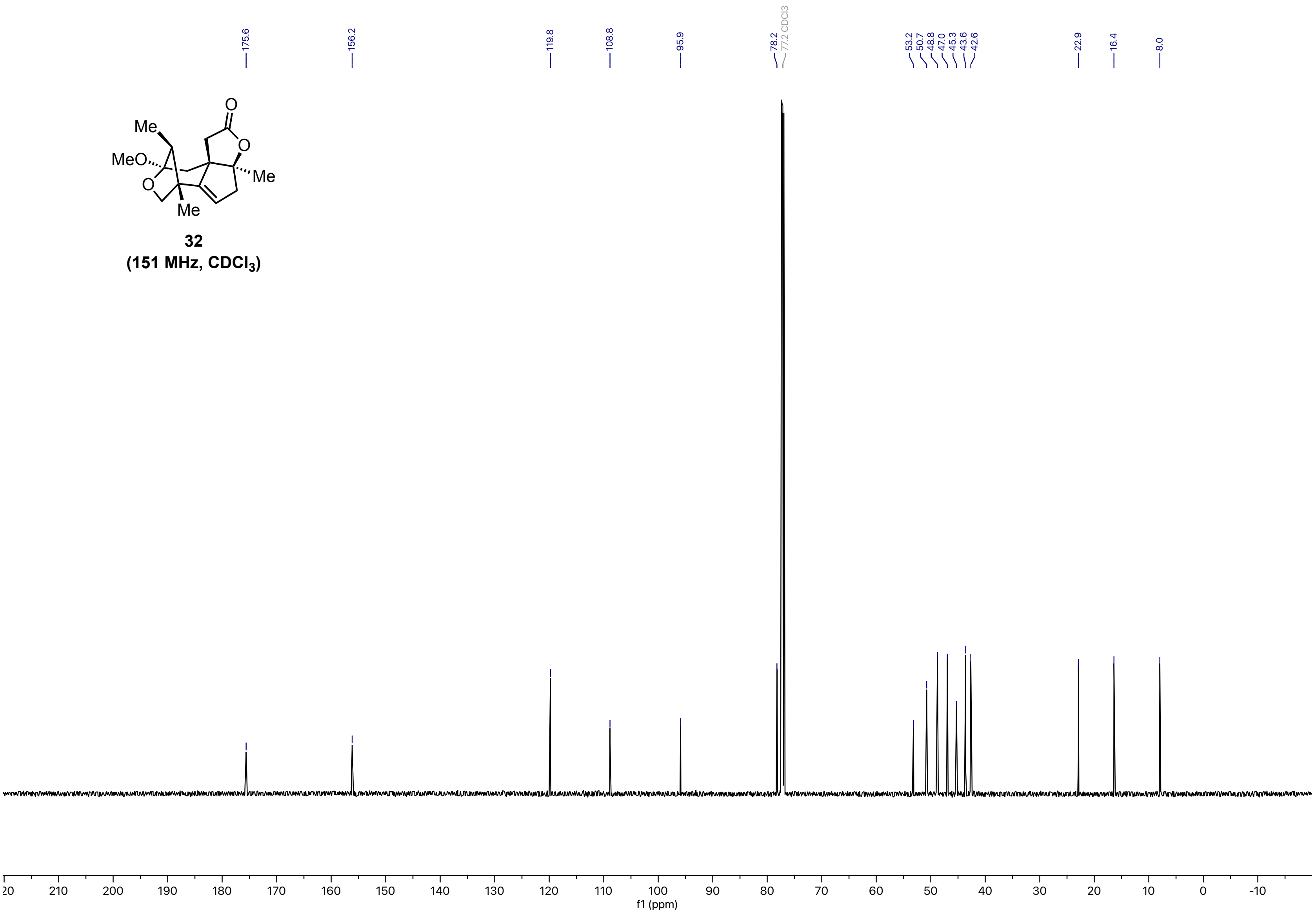
175.6
156.2
119.8
108.8
95.9
78.2
77.2 CDCl3
53.2
50.7
48.8
47.0
45.3
43.6
42.6
22.9
16.4
8.0
Me
MeO
O
O
O
Me
Me
32
(151 MHz, CDCl3)
f1 (ppm)

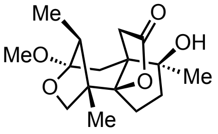


**33**
**(600 MHz, $CDCl_3$)**

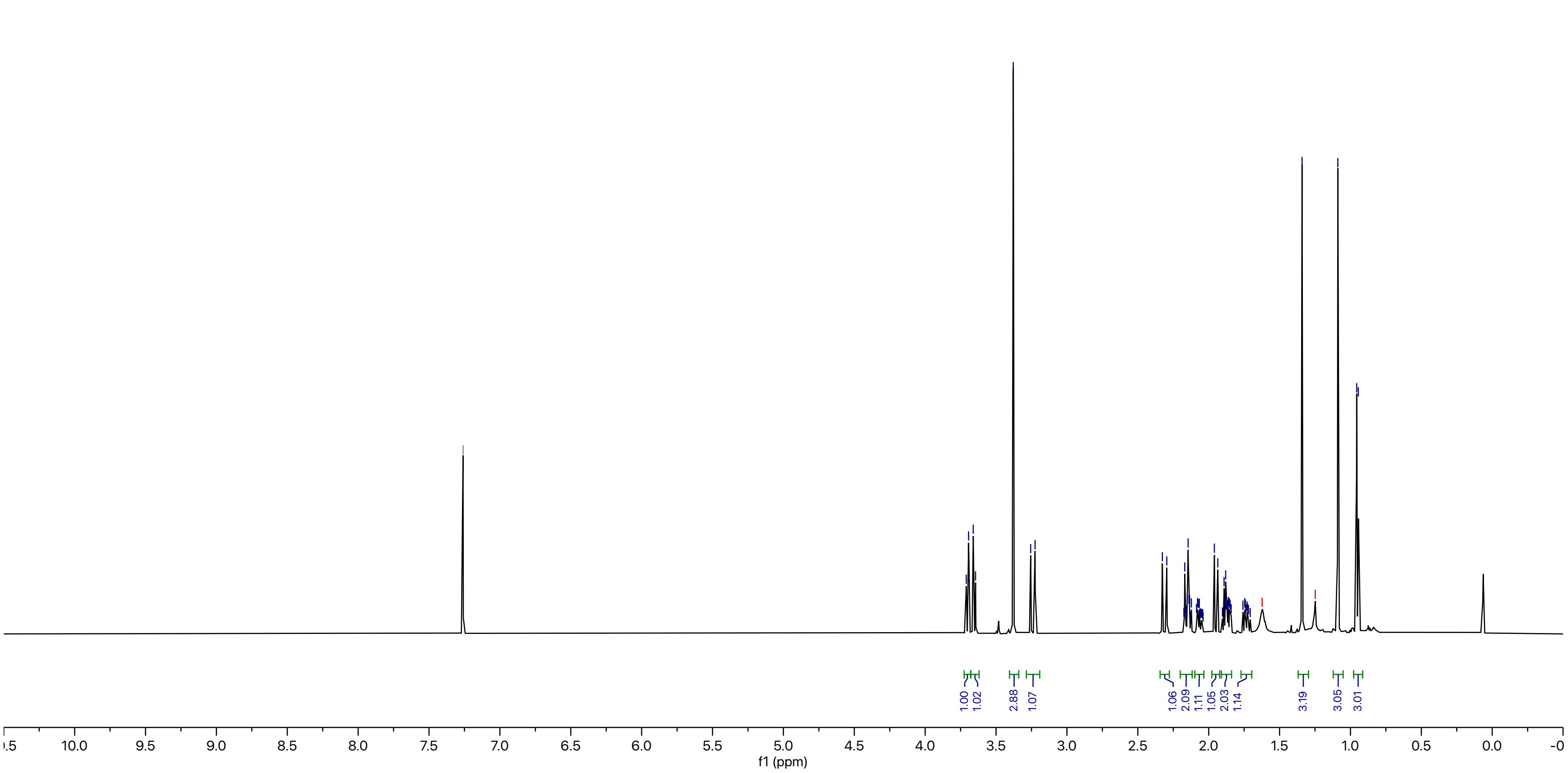

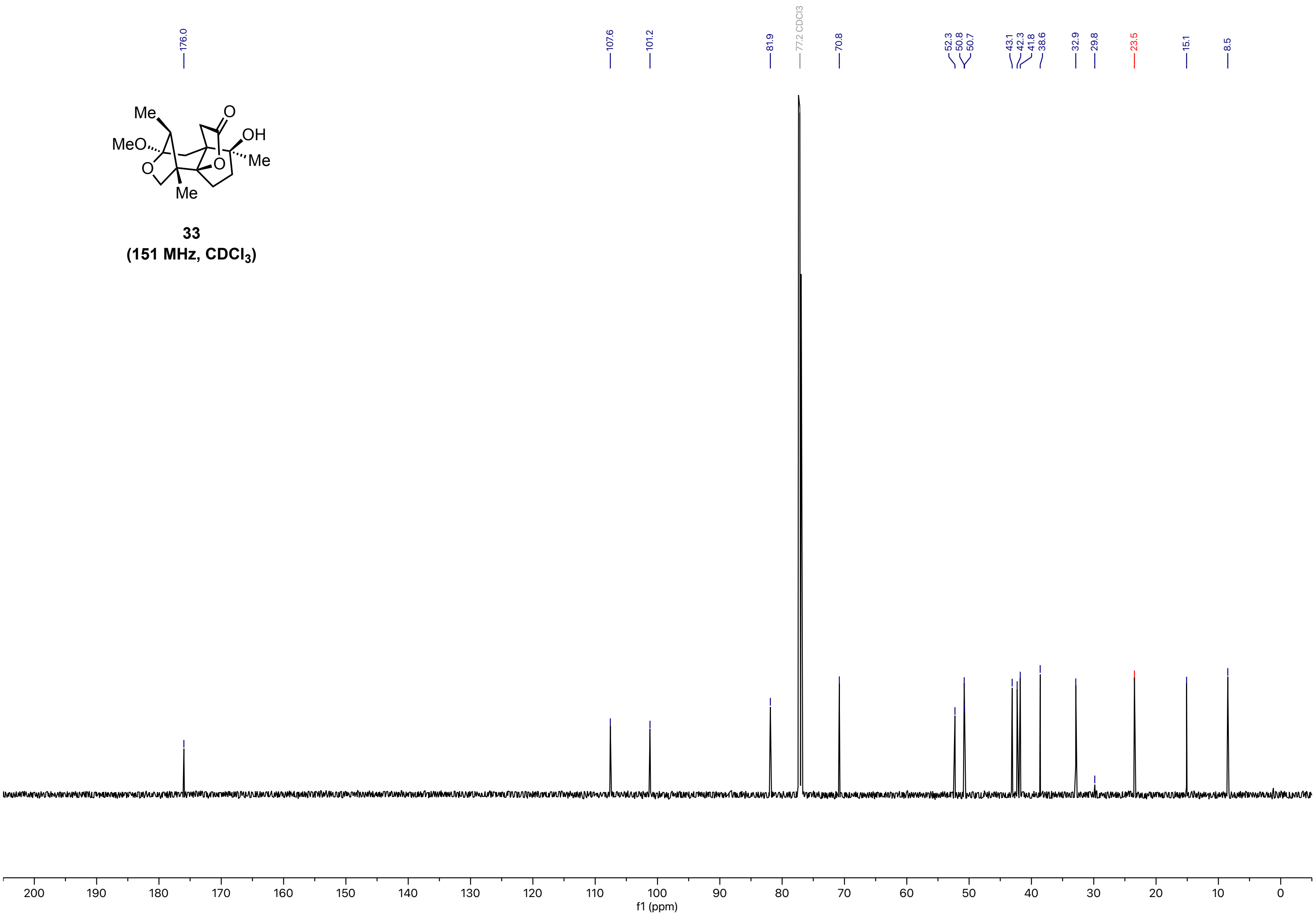
176.0
107.6
101.2
81.9
77.2 CDCl3
70.8
52.3
50.8
50.7
43.1
42.3
41.8
38.6
32.9
29.8
23.5
15.1
8.5
Me
MeO
O
OH
Me
O
O
Me
33
(151 MHz, CDCl3)
200 190 180 170 160 150 140 130 120 110 100 90 80 70 60 50 40 30 20 10 0
f1 (ppm)

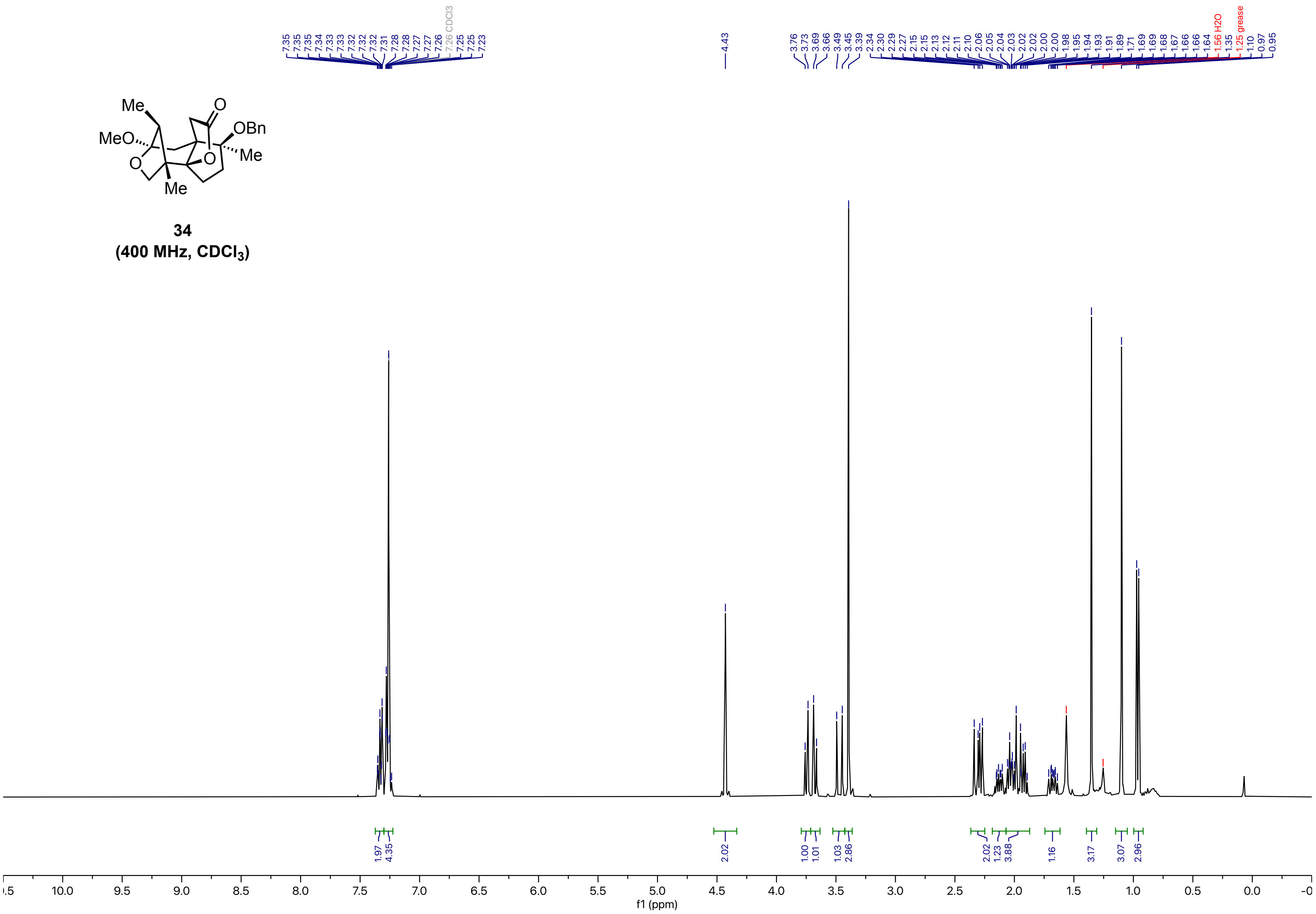

**34**
**(400 MHz, $CDCl_3$)**

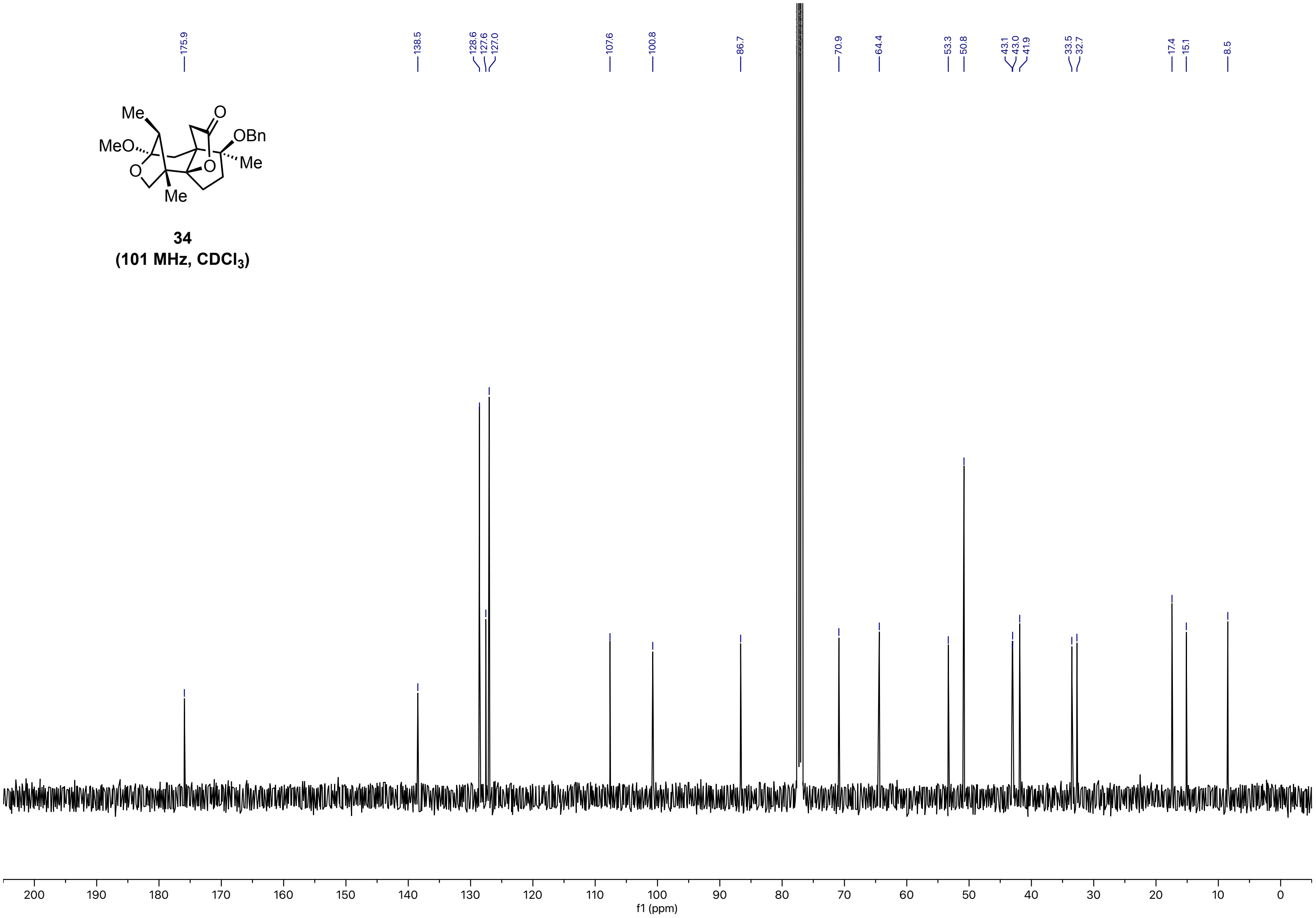

175.9
138.5
128.6
127.6
127.0
107.6
100.8
86.7
70.9
64.4
53.3
50.8
43.1
43.0
41.9
33.5
32.7
17.4
15.1
8.5
Me
MeO
O
OBn
Me
Me
34
(101 MHz, $CDCl_3$)
200 190 180 170 160 150 140 130 120 110 100 90 80 70 60 50 40 30 20 10 0
f1 (ppm)

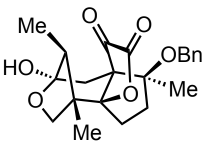


**35**
**(800 MHz, $CDCl_3$)**

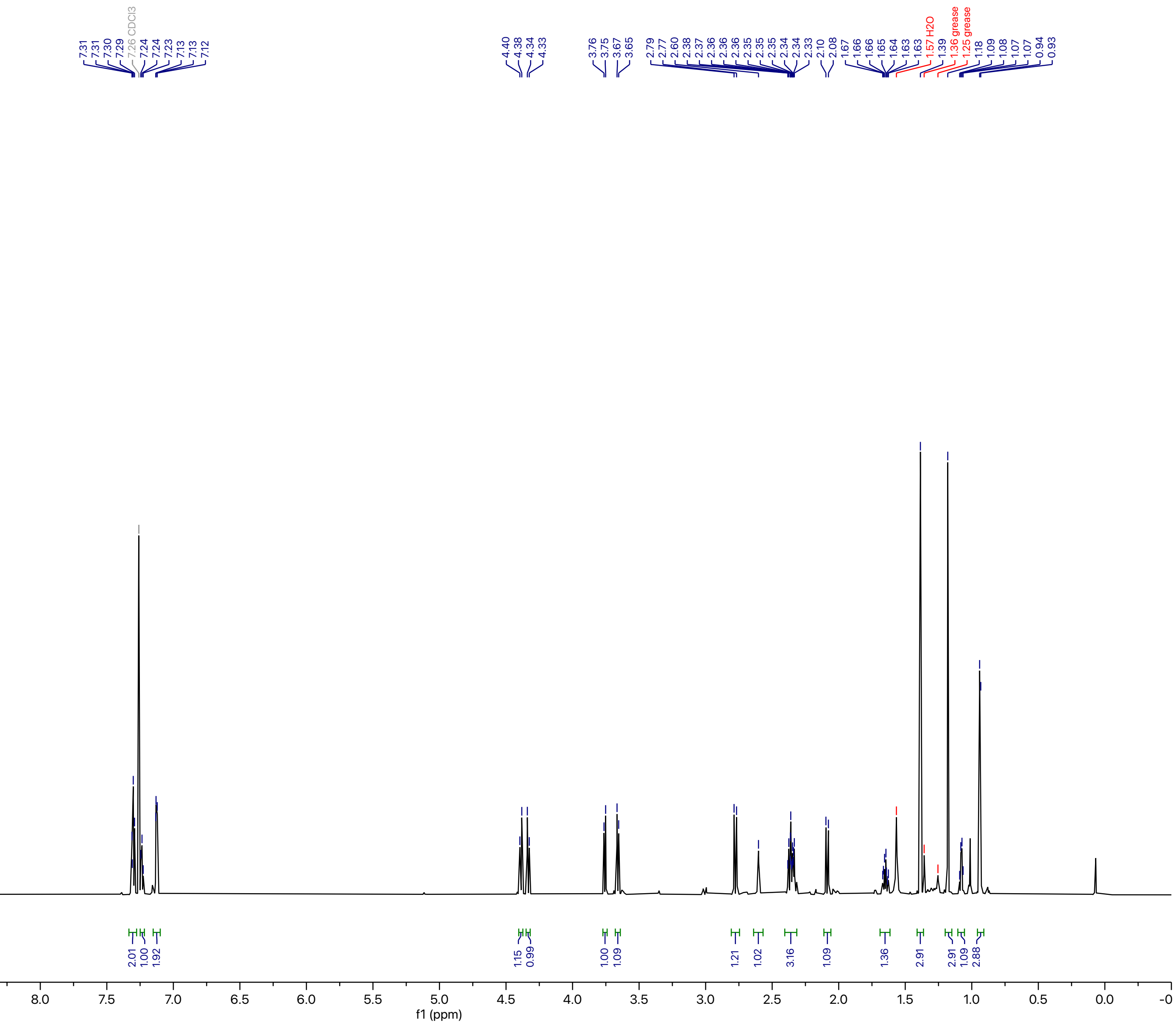

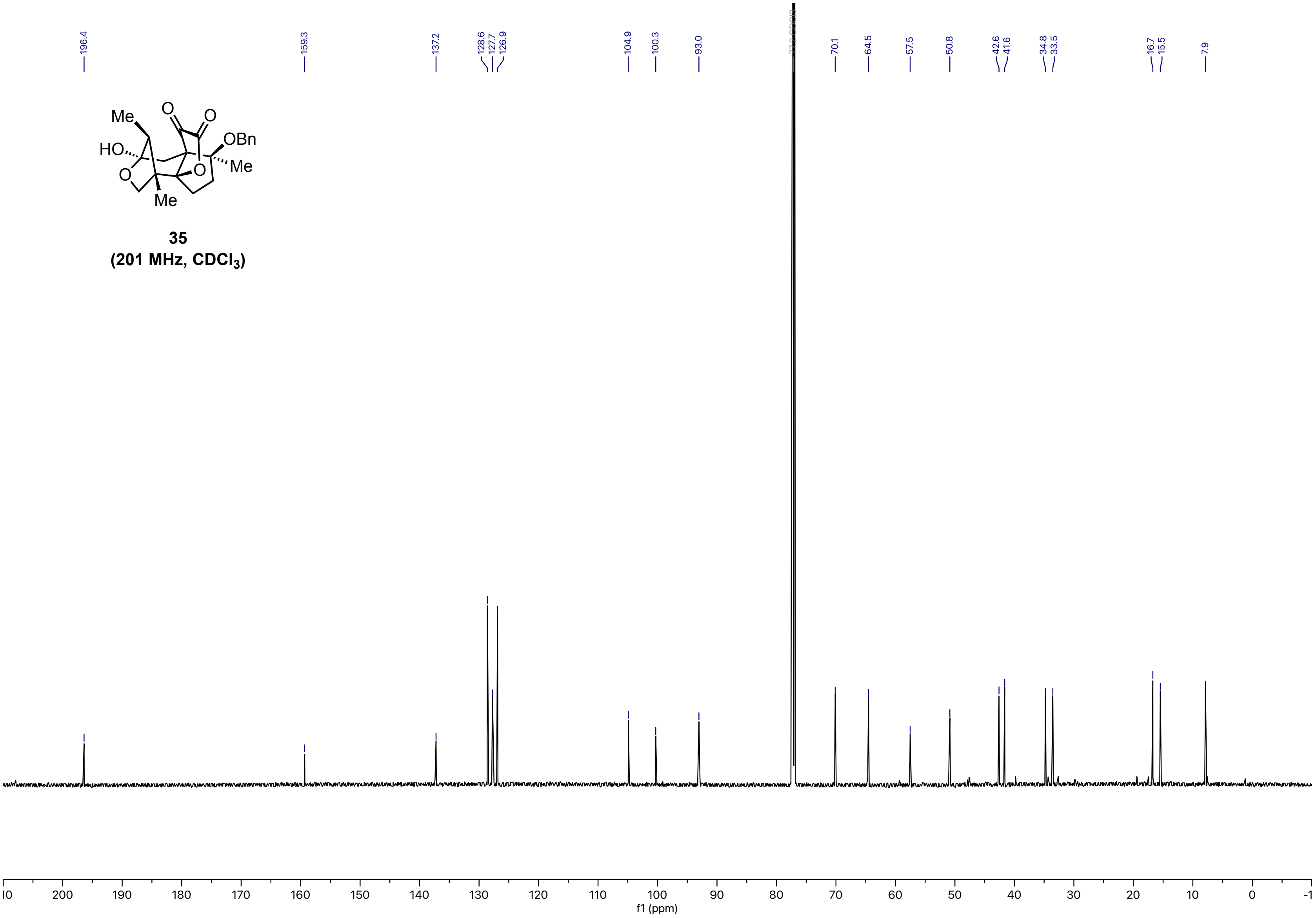


**35**
**(201 MHz, $CDCl_3$)**

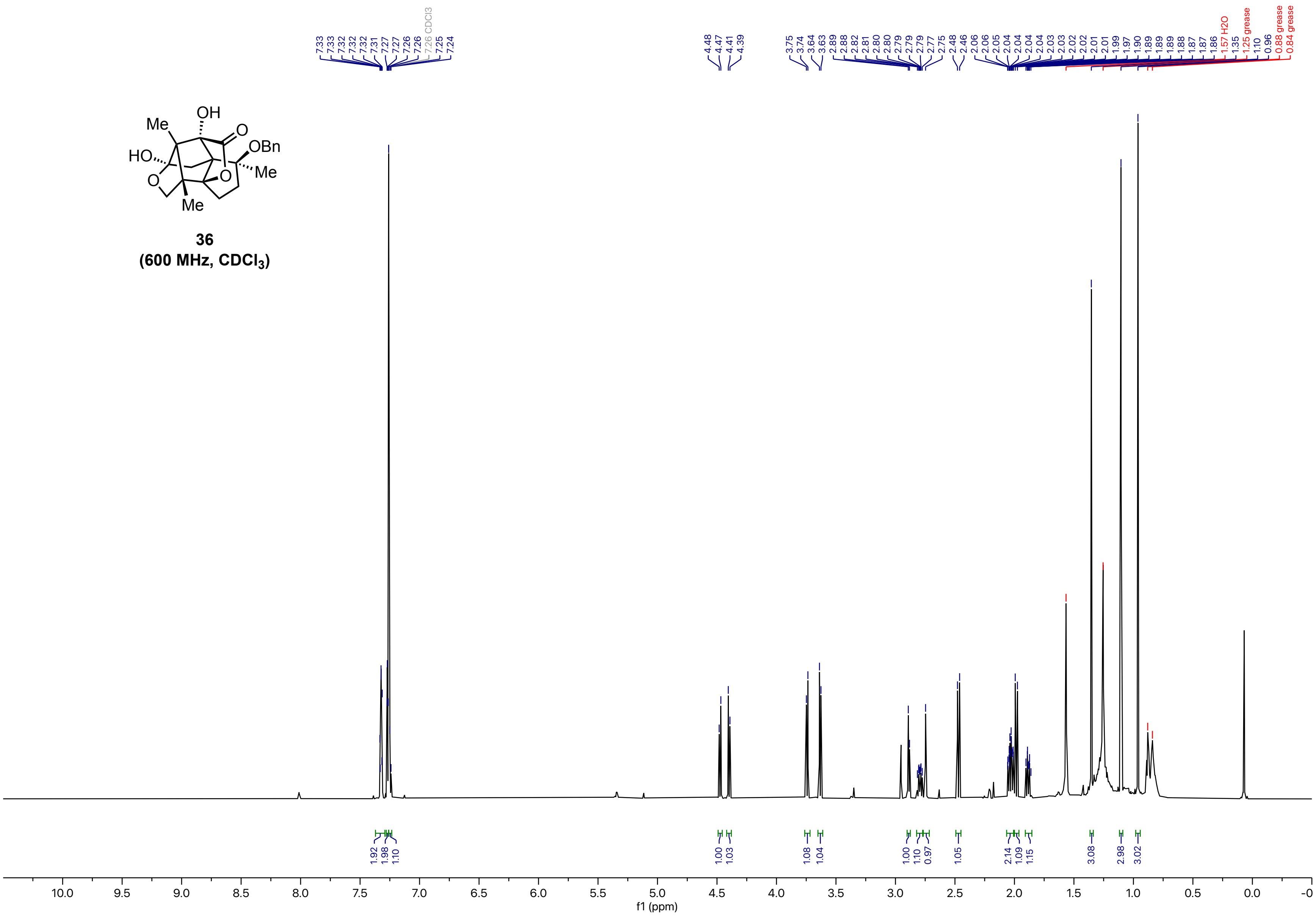
OH
Me
HO
OBn
Me
O
O
Me
36
(600 MHz, CDCl3)
f1 (ppm)

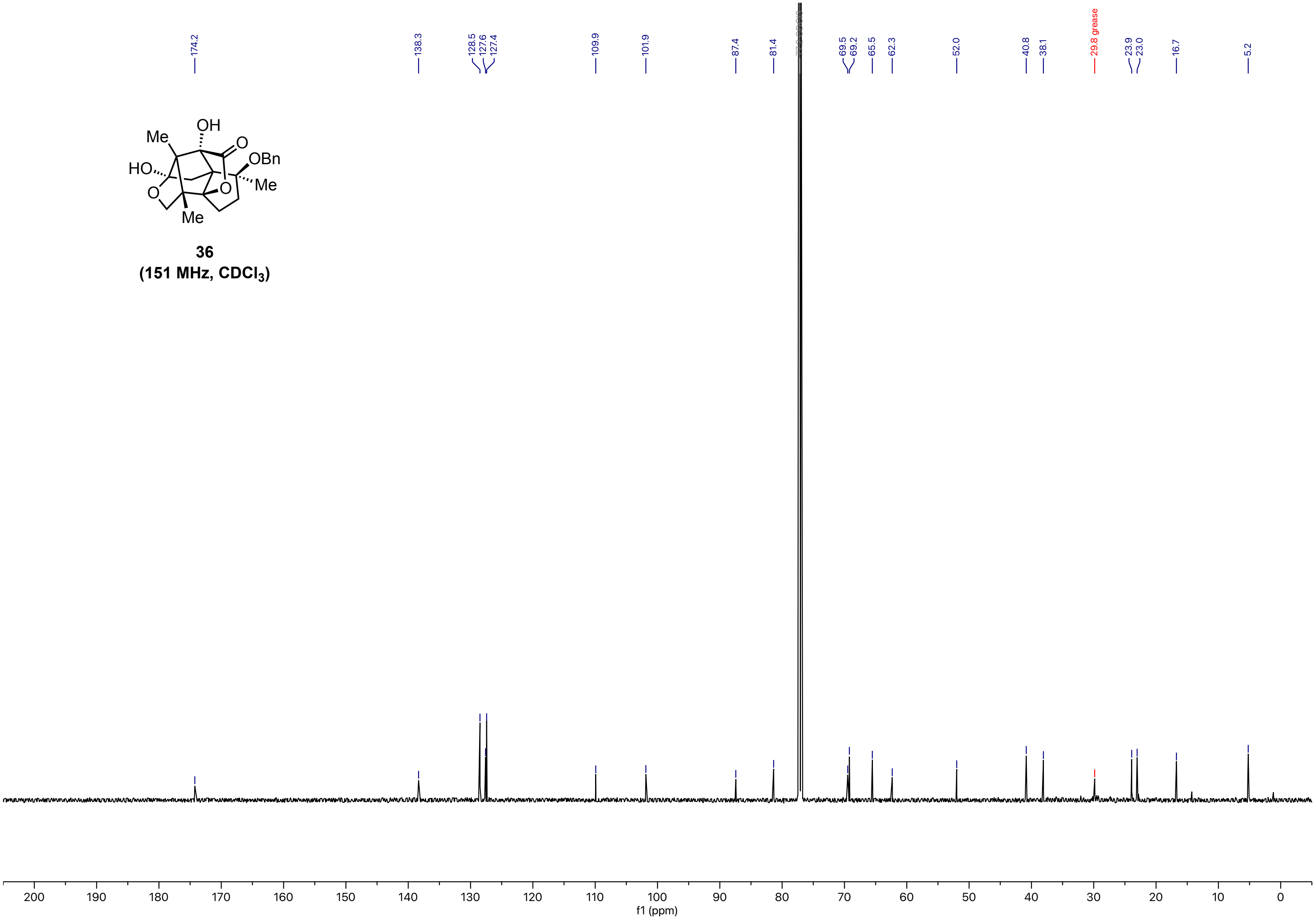


**36**
**(151 MHz, $CDCl_3$)**

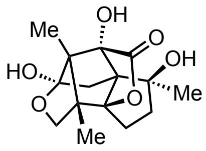


(–)-illisimonin A (**1**)
**(600 MHz, $CD_3OD$)**

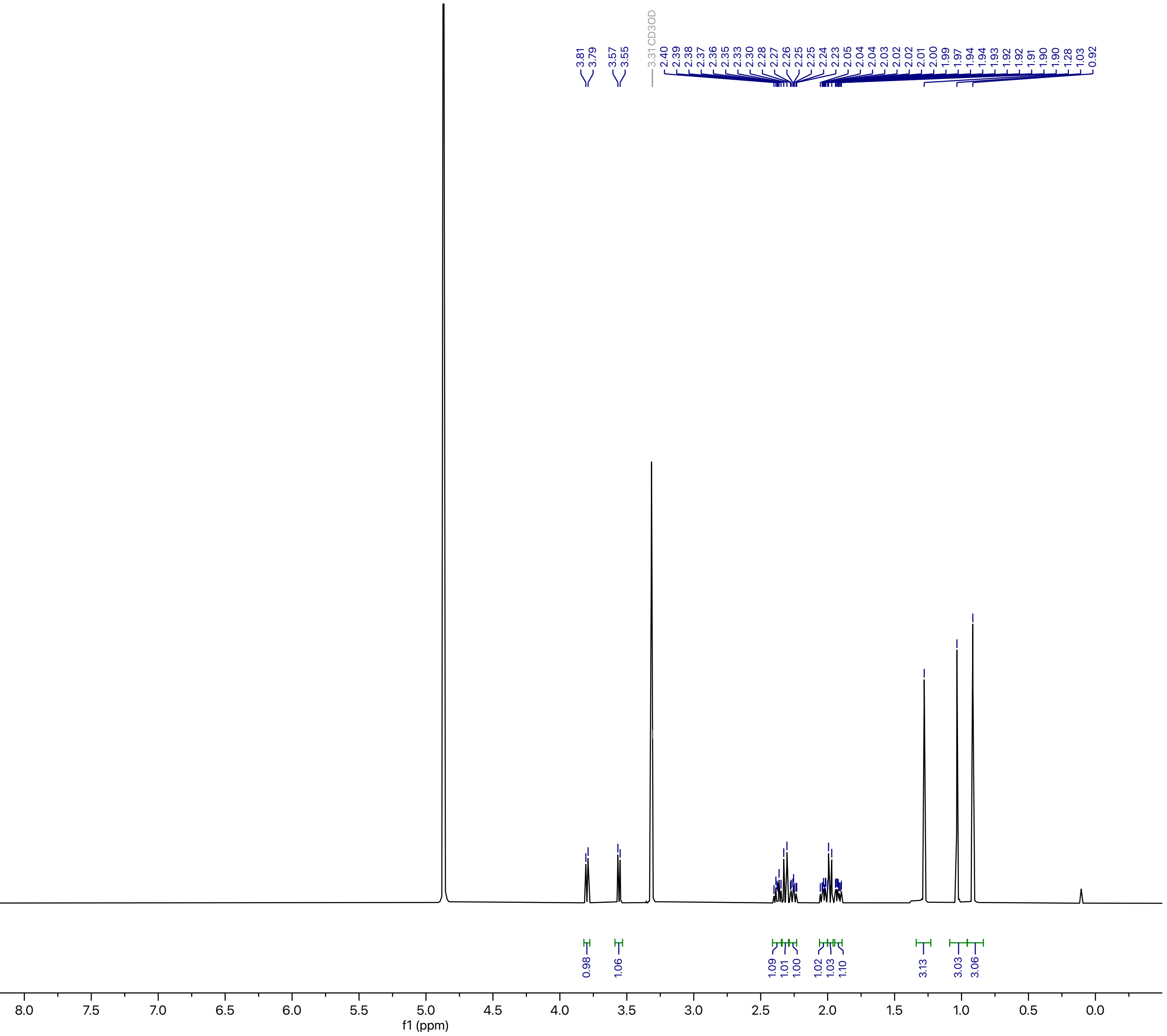

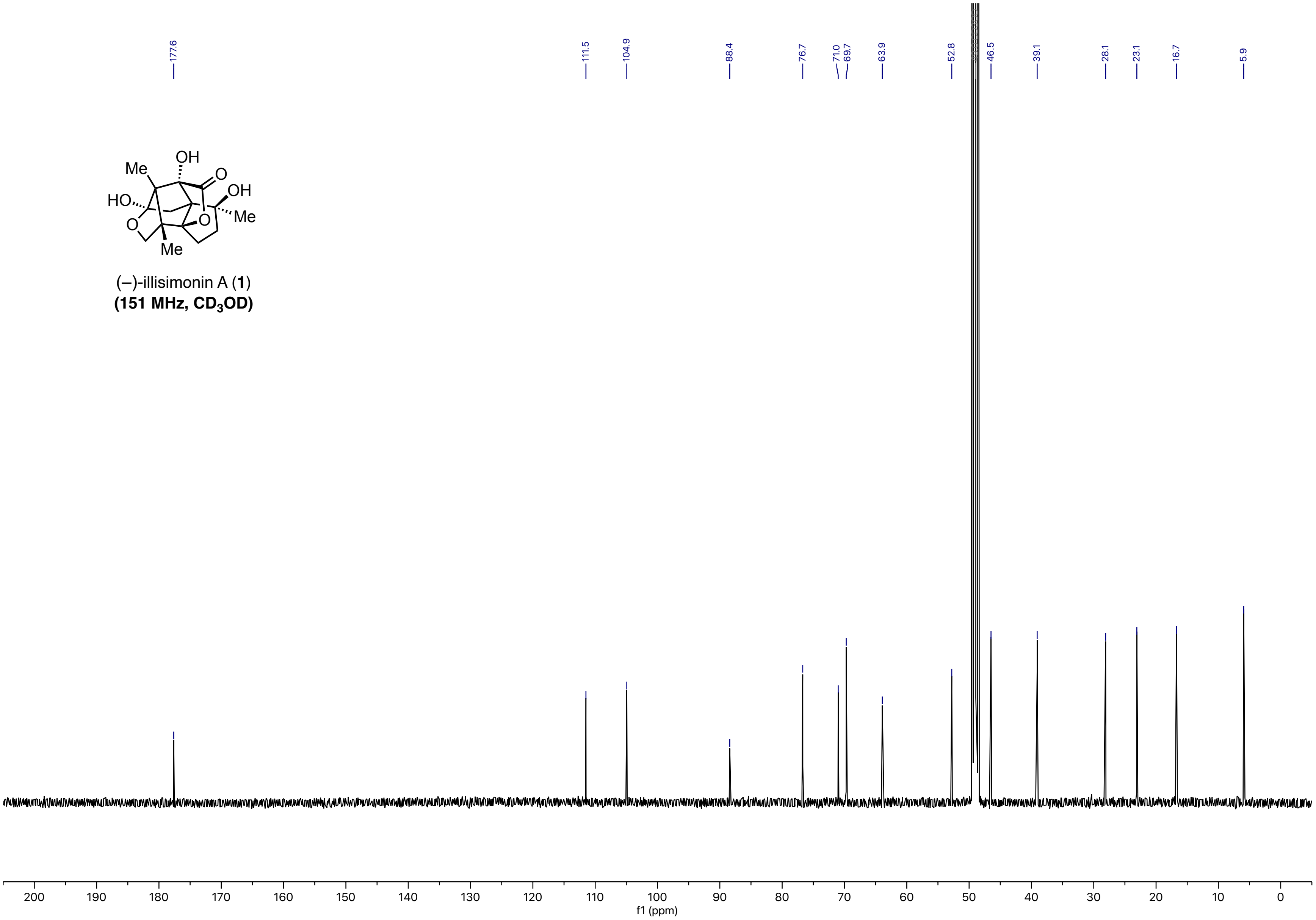
OH
Me
O
HO
OH
O
Me
O
Me
(–)-illisimonin A (1)
(151 MHz, CD3OD)
177.6
111.5
104.9
88.4
76.7
71.0
69.7
63.9
52.8
46.5
39.1
28.1
23.1
16.7
5.9
200
190
180
170
160
150
140
130
120
110
100
90
80
70
60
50
40
30
20
10
0
f1 (ppm)
